# Machine learning reveals targets of *Gnaphalium hypoleucum* DC. flavonoids against rheumatoid arthritis through gut microbiota and anti-inflammation

**DOI:** 10.3389/fimmu.2026.1732859

**Published:** 2026-03-06

**Authors:** Yu-Long Li, Zi-Yong Chu, Ding-Hui Xu, Shu-Yun Wei, Ya-Si Nong, Xiao-Xi Luo, Yi-Jing Wang, Hong Zeng

**Affiliations:** 1 Technology Innovation Cooperation Base of Prevention and Control Pathogenic Microbes with Drug Resistance, School of Basic Medicine, Youjiang Medical University for Nationalities, Baise, China; 2 College of Life Science and Technology, Xinjiang University, Urumqi, China; 3 School of Biomedical and Pharmaceutical Sciences, Guangdong University of Technology, Guangzhou, China

**Keywords:** intestinal microflora, machine learning, molecular docking, rheumatoid arthritis (RA), total flavonoids of *G. hypoleucum* DC. (GHTFs)

## Abstract

**Background:**

Rheumatoid arthritis (RA) is an autoimmune disease characterized by chronic inflammation and gut microbiota dysbiosis. *Gnaphalium hypoleucum* DC. total flavonoids (GHTFs) exhibit anti-inflammatory and immunomodulatory properties.

**Methods and results:**

In this study, we employed an integrated strategy combining machine learning (ML), molecular docking, and molecular dynamics simulations to identify active compounds within GHTFs. The therapeutic mechanisms of these compounds were further investigated using LPS-stimulated RAW264.7 macrophages and a collagen-induced arthritis mouse model. Differential expression analysis identified 2,676 RA-associated genes. A glmBoost + LDA model demonstrated robust diagnostic performance (AUC_train = 0.959; AUC_val ≥ 0.837) and prioritized five key genes (POLB, EGFR, MMP13, VEGFA, and KMT2D). Molecular docking and dynamics simulations confirmed the stable binding of amentoflavone (AF), a primary constituent of GHTFs, to core targets MMP9, MMP13, TOP2A, and ALOX5. *In vitro*, both GHTFs and AF inhibited proliferation, migration, and nitric oxide release in LPS-stimulated RAW264.7 macrophages, and suppressed IL-17, TNF-α, and NF-κB signaling pathways, with AF showing more potent effects (*P* >0.05). *In vivo*, GHTFs treatment reduced clinical arthritis scores by over 40%, alleviated synovial hyperplasia, preserved collagen volume fraction, and lowered serum levels of TNF-α and IL-1β, demonstrating superior overall efficacy compared to AF and methotrexate (*P* >0.05). Gut microbiota analysis revealed that GHTFs enriched beneficial *Lactobacillus* species (e.g., *L. johnsonii, L. intestinalis*), reduced the abundance of pro-inflammatory taxa, and restored microbial metabolic functions.

**Conclusions:**

Collectively, our findings identify GHTFs as a promising therapeutic candidate for RA, ameliorating disease progression through modulation of inflammatory responses and microbiota-mediated immune regulation.

## 1 Introduction

Rheumatoid arthritis (RA) is a chronic condition, systemic inflammatory disease characterized by painful, swollen, and deformed joints, leading to irreversible damage to bone and cartilage. Over time, RA severely impairs patients’ ability to perform daily activities and significantly reduces their quality of life (1). If untreated, it can result in disability or even death. RA has a global prevalence of 0.5–1%, with 0.42% in China, and a high disability rate (2). Despite ongoing research, RA remains incurable, and management primarily relies on drugs such as glucocorticoids, nonsteroidal anti-inflammatory drugs (NSAIDs), and biological inhibitors. However, these treatments often come with adverse side effects, including pain and long-term health risks (1, 3). Several compounds derived from botanicals, including sinomenine, tripterygium glycosides and total peony glucosides, have been approved for the management of RA. Among them, resveratrol has shown notable therapeutic potential in RA, demonstrating efficacy in various pathological conditions (4). This highlights the need for discovering novel, low-toxicity bioactive compounds from traditional herbal medicines to treat RA effectively (5).

Flavonoids in medicinal plants have demonstrated various pharmacological activities, including anti-inflammatory, antibacterial, hypoglycemic, and anticancer effects. Additionally, they can modulate the gut microbiota, thereby enhancing immunity (6–9). *Gnaphalium hypoleucum* DC. (*G. hypoleucum* DC.), a species from the Asteraceae family, is widely distributed in Yunnan, China (10), and is known for its nutritional and medicinal value (11, 12). The primary bioactive compounds in this plant include flavonoids, diterpenoids, volatile oils, and phytosterols (13). *G. hypoleucum* exhibits significant antioxidant, antibacterial, anti-inflammatory, hypotensive, and hypoglycemic activities (13–15). However, the ability of GHTFs (total flavonoids from *G. hypoleucum*) to modulate gut microbiota and enhance immunity in RA treatment remains unclear.

RA pathogenesis involves synovial inflammation, joint destruction, and the activation of immune cells such as mast cells, macrophages, and neutrophils (16), along with the production of matrix metalloproteinases (MMPs) (17, 18). Both genetic and environmental factors, including gut microbiota, contribute to RA development (19). Dysregulation of the gut microbiota has been linked to immune system imbalances, triggering inflammation and potentially initiating RA (20). Targeting the gut microbiota is therefore emerging as a promising immunomodulatory strategy for RA treatment (21).

RA’s pathogenesis is complex, involving multiple targets, signaling pathways, and immune system interactions, making it difficult to identify key therapeutic targets. Traditional drug discovery is slow and expensive, creating a need for more efficient development strategies. Machine learning (ML) has emerged as a powerful tool for bioactivity prediction and structure-activity relationship analysis (22, 23). Recent advances in ML have shown great promise in integrating with natural product research, particularly in predicting bioactivity and uncovering the biological effects of natural compounds (24). ML has also facilitated the design of new drugs by improving bioactivity predictions and computational drug design (25). Numerous studies have demonstrated the potential of ML in discovering natural products with antibacterial, anticancer, and anti-inflammatory properties, such as anthraquinone derivatives (26).

In this study proposes an integrated computational experimental strategy combining ML, molecular docking, and molecular dynamics simulations to identify the most promising bioactive compounds from GHTFs, predict their mechanisms of action, and experimentally validate their therapeutic effects through *in vivo* and *vitro* studies. Particular focus has been given to clarifying the capacity of GHTFs to alter the composition of the gut microbiota and influence host immune responses in RA. This approach establishes a comprehensive framework integrating feature selection, target prediction, structural analysis, and experimental validation, providing a novel strategy for screening RA therapeutic candidates and revealing the pharmacological potential of GHTFs.

## 2 Materials and methods

### 2.1 Preparation of total flavonoids from *G. hypoleucum*

*G. hypoleucum* was harvested from Kunming, Yunnan, China (latitude 25°18′39″N, longitude 102°49′3″E) at an altitude of 2,060 m. The GHTFs were extracted via a previously established method (7). 10kg *G. hypoleucum* was crushed and soaked in 70% ethanol for 30 min and then filtered for purification. After filtration, the residues were washed with 70% ethanol. The pooled filtrates were subsequently processed in a rotary evaporator to remove ethanol, resulting in the acquisition of the 600g crude ethanol extract from *G. hypoleucum*. The crude extract was wet-loaded onto activated AB-8 microporous adsorbent resin and allowed to equilibrate for 1 h. Subsequently, the extract was eluted with 70% ethanol. The collected ethanol eluate was further concentrated and freeze-dried to produce the purified GHTFs. The total flavonoid content was measured via the rutin standard curve method (9). The extracted samples were analyzed via a UPLC–Orbitrap–MS system (UPLC, Vanquish; MS, HFX). HRMS data were recorded on a Q Exactive HFX Hybrid Quadrupole Orbitrap mass spectrometer equipped with a heated ESI source utilizing full-ms-ddMS2 acquisition methods. A total of 37 major components of GHTFs were identified (**Supplementary Table 1**). The samples were analyzed via LC–MS at sanshu Biotechnology (Nantong, China).

### 2.2 ML and computational molecular simulation

#### 2.2.1 Identification of RA-related targets

A total of seven RA-related datasets were obtained from the NCBI GEO database (https://www.ncbi.nlm.nih.gov/geo/). Among them, GSE12021, GSE55235, GSE55457, GSE77298, and GSE89408 were used as training sets, whereas GSE1919 and GSE94519 served as validation sets (27). To mitigate the effects of batch, a multistep normalization pipeline was applied. Surrogate Variable Analysis (SVA) was used to model and adjust for latent confounding factors in the discovery cohort (28). Subsequently, ComBat harmonization was applied to correct residual batch variations via a parametric empirical Bayes framework. Postcorrection, principal component analysis (PCA) revealed improved clustering of interbatch samples in the reduced-dimensional space, thus confirming the success of the data harmonization process (29).

#### 2.2.2 Target prediction of GHTFs

The names of the identified flavonoids were entered into the PubChem compound database to download their three-dimensional structures, which were subsequently saved in SDF format (**Supplementary Table 1**) (14, 30, 31). The structural information of flavonoids was submitted to a target prediction platform. Flavonoids in GHTFs were screened based on gastrointestinal absorption conditions to identify potential targets, such as “High’’ in the Pharmacokinetics project and “Yes’’ in the Drug likeness project (32–37). The filtered GHTFs were input into the SwissTargetPrediction database for active ingredient target prediction (34). The predicted target information was input into the UniProt database for standardization, with ‘‘*Homo sapiens*’’ chosen as the species name, to retrieve the gene name corresponding to the human target (38). The predicted results from all the platforms were subsequently mapped to the UniProt database (https://www.uniprot.org/), with ‘‘*Homo sapiens*’’ selected as the reference species for target standardization. Redundant entries were removed, and the remaining results were consolidated into a nonredundant target set. This final dataset was then used to establish a comprehensive target repository for GHTFs.

#### 2.2.3 Identification of core therapeutic targets for the treatment of RA with GHTFs

A systematic identification of core therapeutic targets of GHTFs for rheumatoid arthritis treatment was performed using an integrated machine learning framework that incorporates multiple algorithms. Using gene expression profiles from the training set, we constructed 127 predictive models based on 12 common ML algorithms, including Lasso, Ridge, Elastic Net (Enet), SVM, glmBoost, plsRglm, stepwise GLM, random forest (RF), GBM, LDA, naive Bayes, and XGBoost. Each algorithm was implemented with multiple hyperparameter configurations and feature subsets to ensure model diversity. Hyperparameter optimization was conducted via 5-fold cross-validation with stratified sampling (39). Model performance was assessed by the area under the ROC curve (AUC), accuracy, and F1 score. To avoid data leakage in stacking, the predicted probabilities of base models were generated via cross-validation and used as input features for a logistic regression meta-learner. Only base models with AUC > 0.945 were retained for stacking.

To ensure robustness in feature ranking, we calculated pairwise correlations among model feature importance vectors. Models with high correlation (Spearman *P* > 0.85) were clustered and down-weighted to reduce redundancy bias. Final target prioritization was based on weighted selection frequency. The performance of the stacking ensemble was compared with that of the best single model and a simple averaging ensemble, confirming the added value of the stacking strategy. Interpretation of the best-performing stacking model was conducted using SHapley Additive exPlanations (SHAP) analysis. SHAP values were estimated via the kernelshap R package, with the prediction function set to output the probability of the “Treatment” class. Global feature importance was defined as the mean absolute SHAP value (mean |SHAP|) per gene across all training samples, thereby avoiding cancellation issues associated with raw SHAP summation. Genes were ranked by mean |SHAP| to identify the most influential therapeutic targets. Core targets were visualized using the ggplot2 package (40).

#### 2.2.4 Molecular docking

The core therapeutic target structures were retrieved from the AlphaFold protein structure database (https://alphafold.ebi.ac.uk/) (41). The ProteinsPlus platform (https://proteins.plus/) was then used to predict the size and location of the potential molecular docking pockets (42). AutoDock was employed to analyze the interactions between pyocyanin and the core targets, following the methodology described by Chu et al. (43). The docking results were visualized using PyMOL, and an energy heatmap of the docking results was drawn (version 3.1).

#### 2.2.5 Molecular dynamics simulation

Molecular dynamics simulations of the protein–ligand complex were performed using GROMACS v2020.6 to evaluate the stability of the binding (44). A rhombic dodecahedron box was built and solvated with TIP3P water, with Na^+^ and Cl⁻ ions added to achieve neutrality and a 0.145 M salt concentration. The simulation parameters were generated using gmx grompp, and then gmx mdrun was used to minimize the energy and remove any unfavourable geometries and energy spikes. Finally, a 100 ns production run was conducted. During the simulation, the RMSD, RMSF, hydrogen bonds, Rg, and SASA were analyzed via the g-rmsd, g-rmsf, g-hbond, g-gyrate, and g-sasa tools (45).

### 2.3 Cell culture

RAW264.7 cells, a mouse leukemic monocyte/macrophage line, were purchased from Kunming Cell Bank, Chinese Academy of Sciences (Kunming, China) and maintained in RPMI-1640 Supplemented with 10% heat-inactivated FBS, penicillin (100 units/mL) and streptomycin (100 μg/mL), and incubated at 37 °C in a humidified atmosphere containing 5% CO_2_.

### 2.4 Transwell assay

After treatment with GHTFs or AF, the cells were seeded into the upper Transwell chamber at a density of 2×10^5^/cells per well in 24-well plates. To test migration ability, 500 μL of RPMI-1640 medium supplemented with 10% FBS was added to the lower Transwell chamber for 24 h. Afterwards, the liquid in the chamber was thrown away, and the chamber was washed twice with PBS to get rid of the rest of the liquid. The 4% formaldehyde used for the fixation of the chamber. The cells in the upper chamber were then removed (46). Subsequent to the chamber being air-dried, it was stained for 15 min in a 0.1% crystal violet solution, which was subsequently washed away with PBS. After air-dried, the chamber was observed under a microscope (Nikon, Tokyo, Japan). Five fields were randomly selected from each chamber and images were captured at 200× magnification. The number of cells that crossed the membrane was determined using ImageJ software, version 1.51 (National Institutes of Health, Maryland, USA). Each test was performed in triplicate.

In order to measure the effects of GHTFs and AF on the migration of LPS-induced RAW264.7 cells, a scratch wound-healing assay was performed. The cells (2 × 10^5^/well) were plated in 6-well plates and cultured for 24 h. Once more than 90% confluence was reached, the cells were subjected to further culture in serum-free medium for a period of 12 h. Subsequently, a superficial abrasion was created on the base of each well, and the cells were treated with LPS and GHTFs or AF. The postscratching images were acquired via a microscope (Nikon, Tokyo, Japan), and the scratch areas were measured and analyzed via ImageJ software version 1.51 (National Institutes of Health). The data are presented as the means ± SDs of three independent experiments (47).

### 2.5 Apoptosis assay

The cells in each group were collected, washed with PBS, and double-stained with PI and Annexin V-FITC in accordance with the manufacturer’s instructions (Beyotime Biotechnology, China). After incubation at 25 °C for 20 min, flow cytometry (Thermo Fisher Scientific, USA) was used to measure the number for further analysis (48).

### 2.6 RA murine model

The collagen-induced arthritis (CIA) model employed in this study closely resembles the various pathological features observed in humans. Eight-week-old (19–21 g) SPF-grade BALB/c female mice were purchased from Guangdong Charles River Laboratory Animal Technology Co. (Guangdong, China). The room temperature ranged from 20-25 °C, the relative humidity ranged from 45–52%, and adaptive feeding was conducted for one week before the experiment. Forty female mice were randomly assigned to five independent groups, control group (n=8), model group (n=8), MTX group (n=8), GHTFs group (n=8), and AF group (n=8). The CIA murine model was constructed according to established methods, which include the use of bovine type II collagen injected in equal parts with Fuchs’ complete adjuvant (49). Following a one-week observation period, the mice that did not develop arthritis successfully underwent a secondary modeling procedure to ensure inclusion. This booster step led to a nearly 100% final success rate. After 20 days, the treatment groups received either MTX (2 mg/kg), AF (100 mg/kg) or GHTFs (100 mg/kg). The control and CIA groups were administered an equal volume of distilled water by continuous gastric gavage for 20 days. On day 40, euthanasia by spinal dislocation under 5% tribromoethanol anesthesia administered intraperitoneally. The blood, joint tissue, and intestinal contents were immediately collected for subsequent analysis.

### 2.7 Pathologic tissue staining and scoring

After 40 d of treatment, the paraformaldehyde-fixed ankle joint tissues were decalcified with EDTA and then cut into 7 μm thick paraffin sections after dehydration, transparency, wax impregnation and embedding. The murine ankle joint tissues were stained according to the instructions of an H&E staining kit (KeyGen Biotech, Nanjing, China) and observed under a light microscope (Tokyo, Japan) (50). The murine ankle joint tissues were stained according to the Masson’s Trichrome Staining Kit (Beyotime, China) instructions, and the number and distribution of collagen fibers in the tissues were observed and analyzed.

### 2.8 Assessment of the gut microbiota composition through 16S rRNA sequencing

Murine fecal samples were collected prior to euthanasia of the mice. The samples were delivered to Shanghai Personal Biotechnology Co., Ltd., for amplicon sequencing on the Illumina NovaSeq 6000 platform (2 × 250 bp).

### 2.9 Statistical analysis

With SPSS (version 27.0) and Origin Pro (version 2024b) software, each experiment was repeated independently three times, and the data were subjected to statistical analysis and are presented as the means ± SDs. Prior to comparative analyses, the normality of data distribution was assessed using the Shapiro–Wilk (S-W) test. For data conforming to a normal distribution, intergroup and intragroup comparisons were evaluated via analyses of variance (ANOVAs) followed by the Duncan test. The relationships between two variables were examined through Pearson correlation analysis. A *P* value of < 0.05 was considered to indicate a statistically significant difference.

## 3 Results

### 3.1 ML and computational molecular simulation analysis

#### 3.1.1 Results of the screening of GHTFs

The information on the GHTFs obtained from our previous work was imported into the Swiss ADME database, and a total of 15 lead compounds were identified according to the method described in section 2.2.2. Their numbers are MOL01, MOL02, MOL03, MOL04, MOL05, MOL06, MOL07, MOL08, MOL09, MOL10, MOL11, MOL12, MOL13, MOL14, and MOL15 (**Table 1**).

**Table 1:** Results of the screening of flavonoids.

Compound no.	Compounds	Formula	Molecular weight
MOL01	Gardenin B	C_19_H_18_O_7_	358.11
MOL02	Corymbosin	C_19_H_18_O_7_	358.11
MOL03	5,4’-Dihydroxy-3,6,7,3’ tetramethoxyflavone	C_19_H_18_O_8_	374.10
MOL04	Ayanin	C_18_H_16_O_7_	344.09
MOL05	3,5-dihydroxy-6,7,8, 4’-tetramethoxyflavone	C_19_H_18_O_8_	374.10
MOL06	Hymenoxin	C_19_H_18_O_8_	374.10
MOL07	Cirsilineol (4’,5-Dihydroxy-3’,6,7-trimethoxyflavone)	C_18_H_16_O_7_	344.09
MOL08	Disporopsin	C_16_H_14_O_6_	302.08
MOL09	Natsudaidain	C_21_H_22_O_9_	418.13
MOL10	5-Hydroxy-3,7,3’,4’-tetramethoxyflavone (Retusin)	C_19_H_18_O_7_	358.11
MOL11	Kumatakenin	C_17_H_14_O_6_	314.08
MOL12	Cupressuflavone	C_30_H_18_O_10_	538.09
MOL13	Amentoflavone	C_30_H_18_O_10_	538.09
MOL14	Apigenin	C_15_H_10_O_5_	270.05
MOL15	Luteolin	C_15_H_10_O_6_	286.05

#### 3.1.2 Identification of RA-related targets

**Figure 1:**
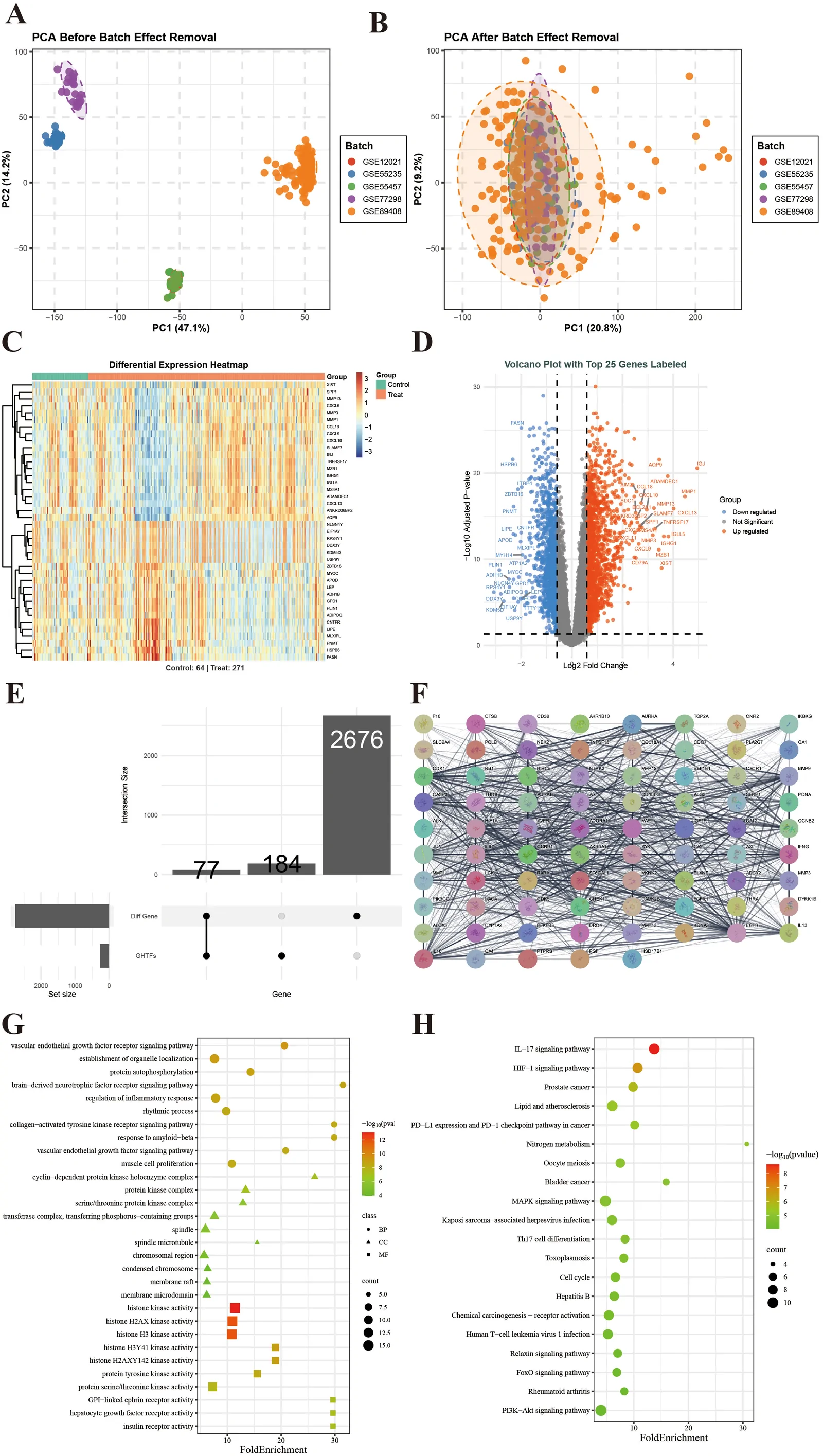
Preliminary identification of potential therapeutic targets for RA. **(A)** PCA reveals the separation of the datasets before batch correction. **(B)** Batch-corrected PCA reveals dataset integration and reduced batch effects. **(C)** Heatmaps displaying the expression patterns of DEGs across samples. **(D)** Volcano plot displaying DEGs identified by logFC and statistical significance. **(E)** UpSet graph illustrating genes overlapping between GHTFs and RA. **(F)** The PPI network displays interactions among the overlapping genes. **(G)** The histogram displays the top 10 enriched terms in each GO category (BP, CC, and MF) ranked by smaller FDR values. **(H)** Bubble diagram displaying the top 20 KEGG pathways enriched in reverse order of FDR values.

To eliminate batch effects and improve comparability between samples, five gene expression datasets (GSE12021, GSE55235, GSE555457, GSE77298, and GSE89408) were integrated and uniformly normalized. PCA indicated that normalization led to a more reasonable sample distribution, with clearer clustering separation between the RA and control groups (**Figures 1A, B**). Differential expression analysis revealed a total of 2,753 genes whose expression significantly differed between RA and normal tissues. The expression patterns of these genes have been visualized using the volcano plots and heatmaps, which have suggested their potential roles in the onset and progression of RA (**Figures 1C–E**).

#### 3.1.3 Identification of therapeutic targets associated with RA in GHTFs

By intersecting predicted GHT targets with RA-related targets we identified 77 potential therapeutic targets that may mediate the therapeutic effects of GHTFs on RA (**Figures 1E, F**). Functional characterization via GO and KEGG enrichment analyses provided comprehensive insights into the underlying molecular mechanisms (**Figures 1G, H**). GO and KEGG enrichment analyses revealed that GHTFs-RA intersecting targets were significantly enriched in various biological processes and signaling pathways closely related to immunity and inflammation. GO analysis revealed that these targets were primarily implicated in various processes such as “regulation of inflammatory response”, “vascular endothelial growth factor signaling pathway”, and “collagen-activated tyrosine kinase receptor signaling pathway”, suggesting their potential role in regulating immune responses, participating in joint cartilage destruction and synovial fibrosis, and treating joint inflammation.

At the molecular function and cellular component levels, the targets were enriched in “protein tyrosine kinase activity”, and “protein serine/threonine kinase activity”, further supporting their involvement in immune cell activation and tissue inflammation. KEGG pathway analysis revealed enrichment of key inflammatory pathways, including RA, the IL-17 signaling pathway, and Th17 cell differentiation, as well as immune regulation pathways, such as the MAPK and PI3K–Akt signaling pathways. These results suggest that GHTFs might attenuate RA progression by regulating inflammatory responses, reducing synovial vascular proliferation, inhibiting bone erosion, and protecting immune homeostasis.

#### 3.1.4 Identification of core therapeutic targets in RA treated with GHTFs

**Figure 2:**
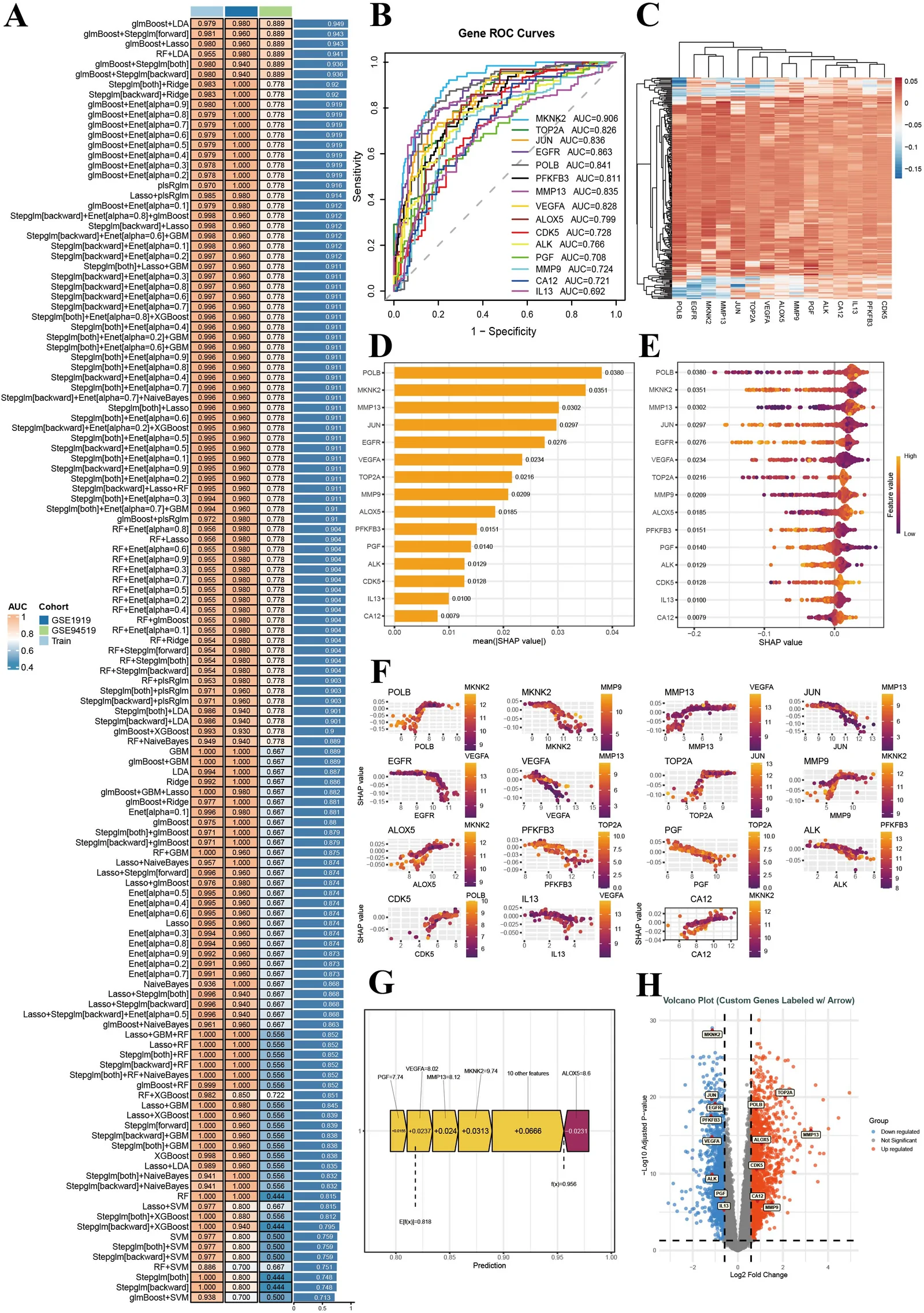
Identification of key therapeutic targets in RA. **(A)** Heatmap of model performance showing AUC values across different cohorts. The models are listed on the left, the AUC values are listed on the right, and the colors indicate the cohort origin. **(B)** ROC curves illustrating the predictive performance of six key genes. The X-axis denotes the false positive rate; the Y-axis denotes the sensitivity. The AUC values represent the prediction accuracy. **(C)** Cumulative SHAP contribution curve. **(D)** SHAP summary of feature importance for model interpretation. **(E)** SHAP beeswarm plot showing the impact of features on model predictions. **(F)** SHAP value distribution: key gene contributions to prediction visualized through SHAP scatter plots. **(G)** SHAP force plot showing the contribution of features to model predictions. **(H)** Volcano plot showing DEGs, with logFC on the X-axis and -log10 (p value) on the Y-axis. Key genes are annotated.

Among all the model combinations tested, the glmBoost + LDA model exhibited the most consistent and robust diagnostic performance, achieving an AUC of 0.959 in the training set and maintaining high generalizability, with AUCs of 0.883 and 0.837 in the GSE1919 and GSE4619 validation cohorts, respectively (**Figure 2A**). SHAP analysis revealed that this model relies primarily on five genes—*Polb*, *Egfr*, *Mmp13*, *Vegfa*, and *Mknk2*—which together account for most of the model’s predictive power. The SHAP summary plot (**Figures 2D, E**) shows that higher expression levels of these genes contributed more positively to disease classification, with POLB and EGFR showing particularly consistent effects across samples. Heatmap visualization of these genes (**Figure 2C**) revealed clear expression segregation between the disease and control groups, supporting their discriminatory ability at the transcriptome level. ROC analysis further confirmed their diagnostic value as single features, with AUCs exceeding 0.80 for all five genes (**Figure 2B**). SHAP interaction analysis (**Figure 2F**) demonstrated notable synergistic effects, particularly between POLB and MMP13 and between EGFR and VEGFA, suggesting cooperative regulatory influence within the model. A representative SHAP waterfall plot (**Figure 2G**) illustrates how additive contributions from these key genes drove the model toward high-confidence disease prediction. Differential expression analysis (**Figure 2H**) corroborated these findings, as all five SHAP-prioritized genes were significantly upregulated in disease samples. Collectively, these results demonstrated that the glmBoost + LDA model not only achieved excellent classification accuracy but also provided biologically interpretable insights, enabling the identification of a small panel of highly informative and differentially expressed genes that may serve as key targets of GHTFs in the treatment of RA.

#### 3.1.5 Molecular docking and molecular dynamics simulation

**Figure 3:**
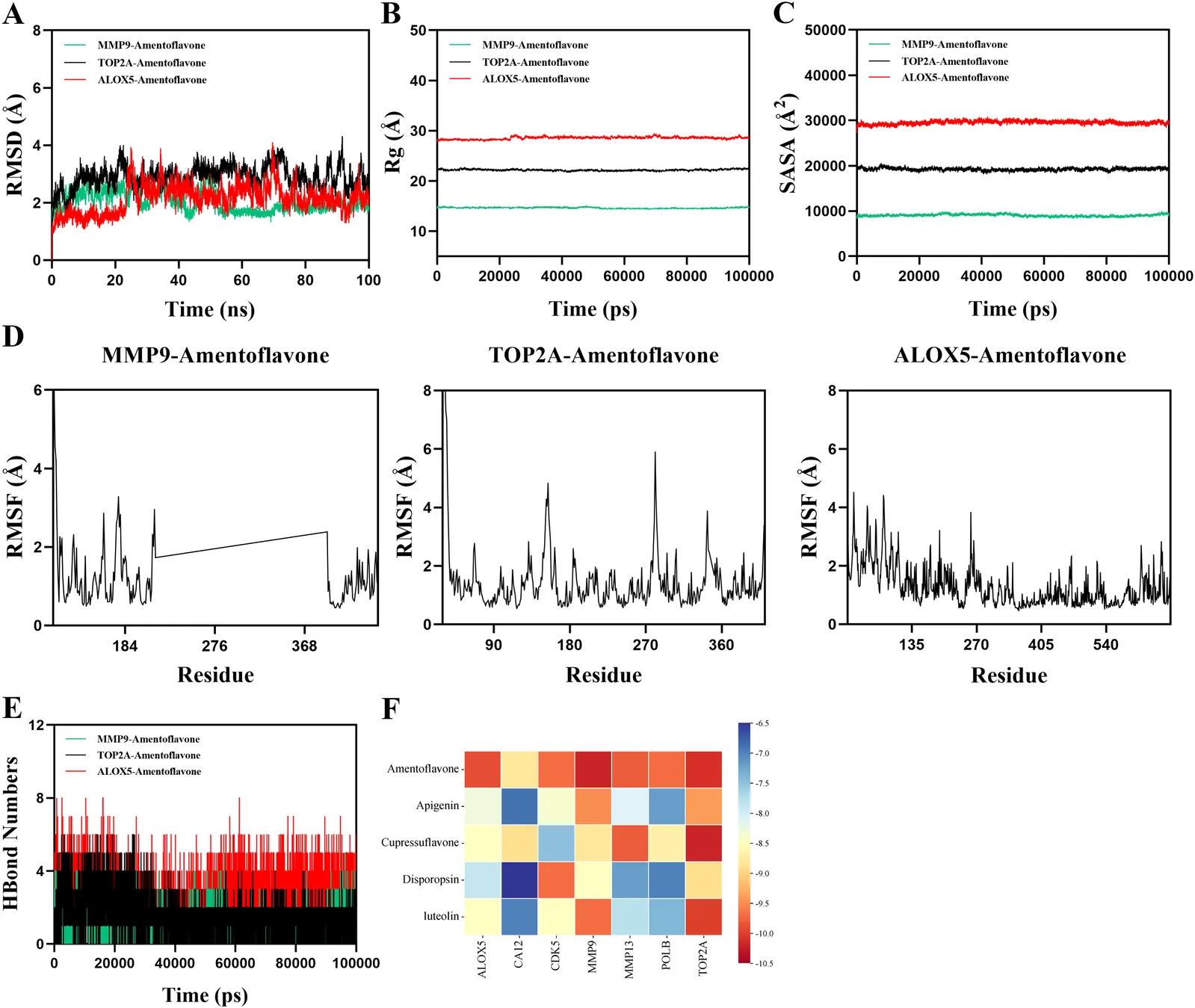
Molecular dynamics simulation. **(A)** RMSD values of protein–ligand complexes over time. **(B)** Rg values of protein–ligand complexes over time. **(C)** SASA values of protein–ligand complexes over time. **(D)** HBond values of protein–ligand complexes over time. **(E)** RMSF values of protein–ligand complexes. **(F)** Heatmap of the molecular docking binding energy of ingredients to core targets.

**Figure 4:**
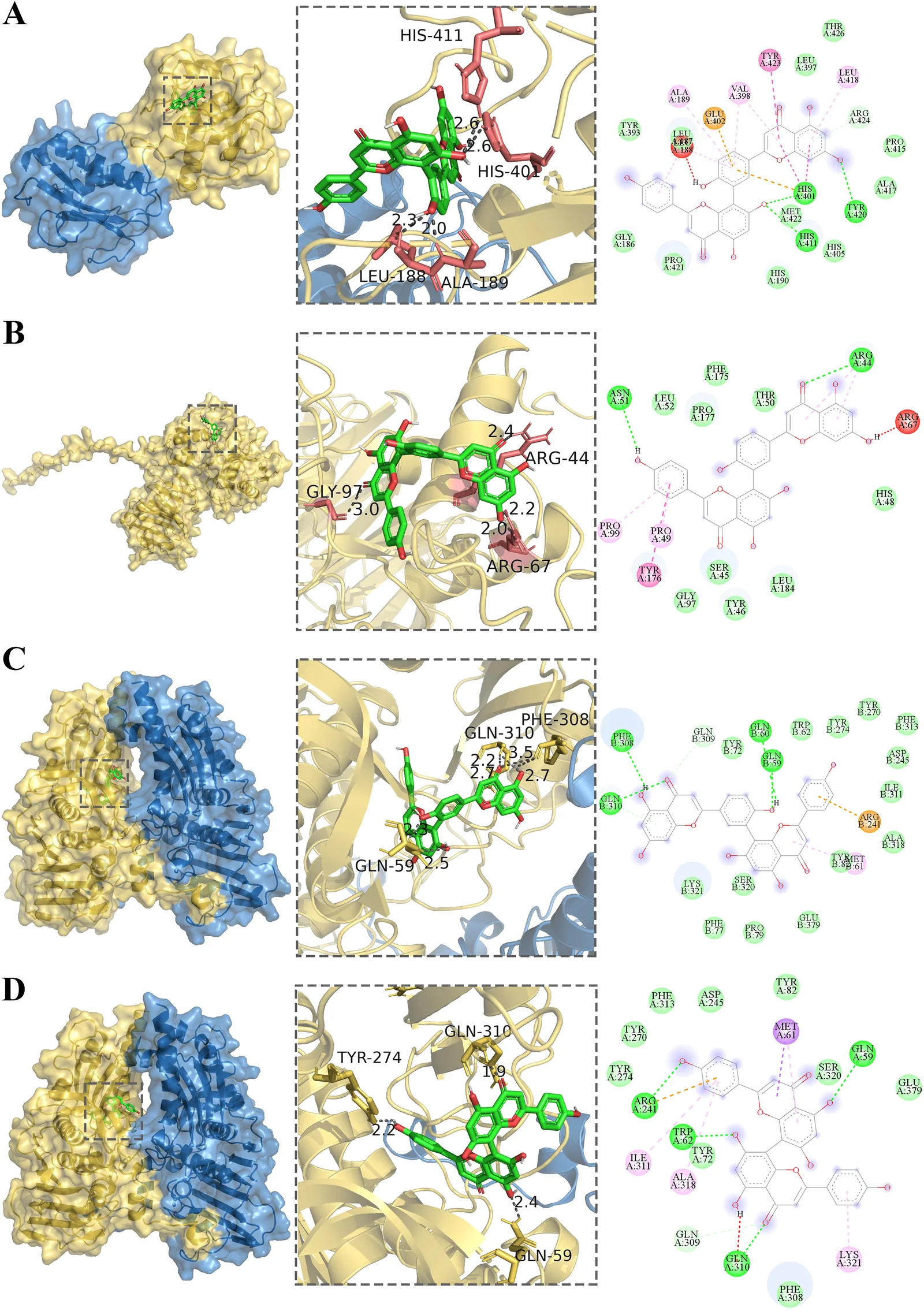
Molecular docking validation of key therapeutic targets for RA. **(A)** MMP9 and AF interaction graph. **(B)** MMP13 and AF interaction graph. **(C)** TOP2A and AF interaction graph. **(D)** TOP2A and CF interaction graph.

Molecular docking analysis was used to validate the interactions between fifteen flavonoids and seven core therapeutic targets implicated in the treatment of RA, ALOX5, CA12, CDK5, MMP9, MMP13, POLB, and TOP2A. Among these, five flavonoids exhibited strong binding affinities toward all seven targets, with binding energies below -6.5 kcal/mol (**Figure 3F**; **Supplementary Figure 1**), indicating stable and spontaneous intermolecular interactions. Visualization of the docking conformations demonstrated that the compounds occupied the active binding pockets of these target proteins in a stable manner. In addition, AF primarily conjugated the amino acid residues HIS-401, LEU-188, and ALA-189 of MMP9 and the amino acid residues ARG-44, ARG-69, and GLY-97 of MMP13 (**Figures 4A, B**). AF also bound to residues PHE-308, GLN-310, and GLN-59 of TOP2A. Similarly, CF bound to GLN-310, TYR-274, and GLN-59 of TOP2A (**Figures 4C,D**). The complexes with the lowest energy savings, AF-MMP9, AF-TOP2A, and AF-ALOX5, were subsequently selected for molecular dynamics simulations.

The simulation results revealed that the MMP9-mentoflavone complex system reached equilibrium after 50 ns and ultimately fluctuated around 1.8 Å. The TOP2A-mentoflavone complex system fluctuated stably between 25 ns and 70 ns, and after 70 ns, it fluctuated, but overall, it fluctuated below 2.8 Å. The ALOX5-mentoflavone complex system reached equilibrium after 75 ns and ultimately fluctuated around 2 Å, indicating high conformational stability (**Figure 3A**). Rg fluctuations suggested conformational changes; however, the MMP9-AF, TOP2A-AF, and ALOX5-AF complexes exhibited relatively stable fluctuations during the simulation. These observations indicated that the small molecule–target protein complexes did not undergo substantial expansion or contraction during movement. SASA remained stable, indicating minimal structural impact from ligand binding (**Figures 3B, C**). The RMSF can be used to indicate the degree of flexibility of amino acid residues in proteins. The RMSF values of the MMP9-AF, TOP2A-AF, and ALOX5-AF complexes are relatively low (mostly below 3 Å), indicating that these complexes have low flexibility and high stability (**Figure 3D**). Hydrogen bond analysis revealed one stable hydrogen bond, indicating strong ligand–protein interactions (**Figure 3E**).

Taken together, these structural observations suggest a direct binding of AF to MMP9, TOP2A, and ALOX5, thereby contributing to the progression of RA. These targets may represent key mediators in the treatment of RA with GHTFs and provide potential clinical approaches.

### 3.2 GHTFs and AF inhibit LPS-induced proliferation, apoptosis, and migration in RAW264.7 macrophages

**Figure 5:**
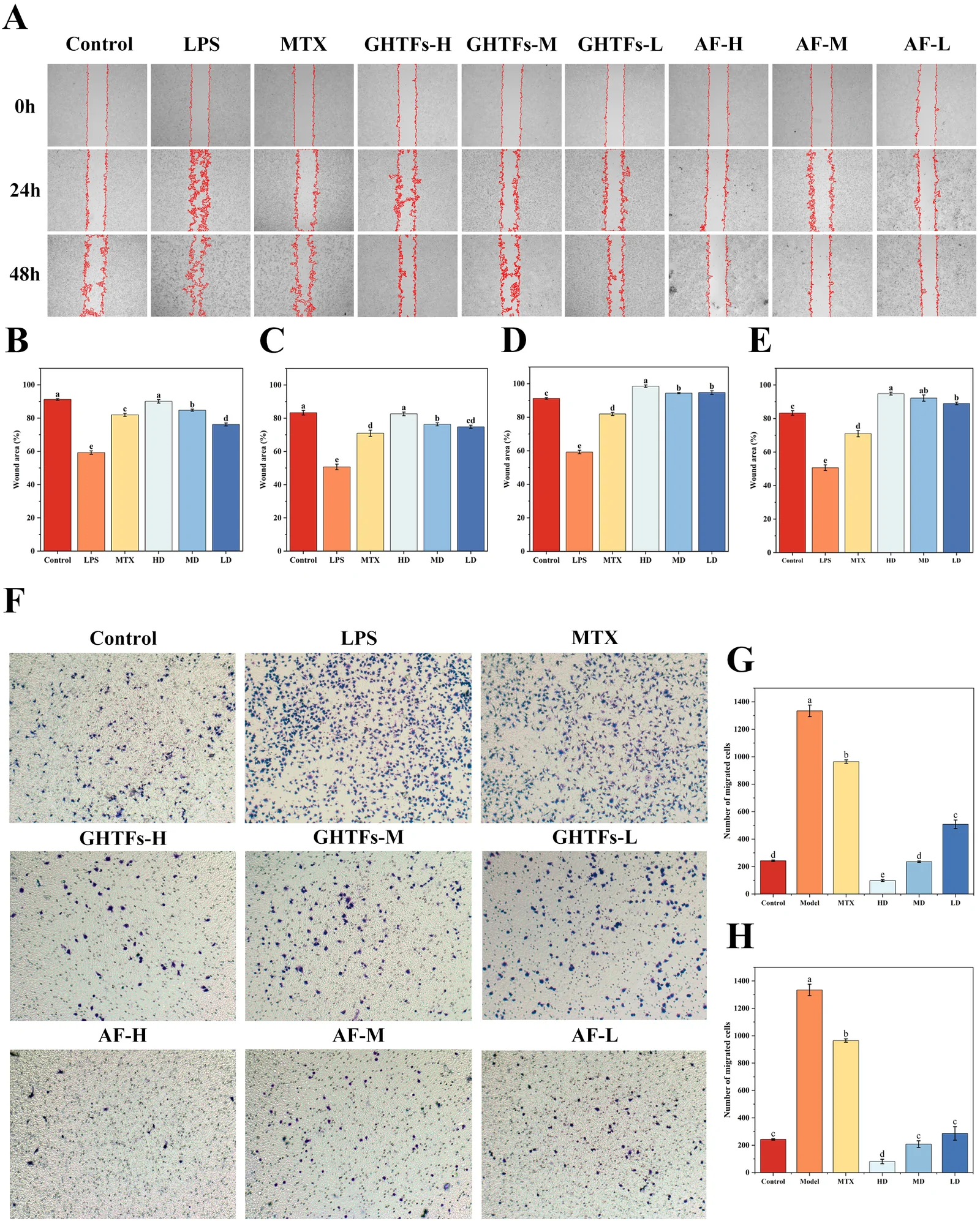
GHTFs/AF suppress the migration of LPS-induced RAW264.7 cells. **(A)** The antimigratory effects of GHTFs and AF (125, 62.5, and 31.25 μg/mL) were assessed via a scratch wound-healing assay. Wound closure was monitored at 0, 24, and 48 h under a microscope (100× magnification). Migration inhibition was quantified by measuring the percentage reduction in the scratch width compared with the initial wound area. Representative images illustrate the effects of **(B)** 24 h of treatment with GHTFs, **(C)** 48 h of treatment with GHTFs, **(D)** 24 h of treatment with AF, and **(E)** 48 h of treatment with AF. The data are presented as the means ± SEMs from at least three independent experiments. **(F)** Further validation was performed via a Transwell migration assay after 24 h of treatment. The migrated cells were fixed, stained, and visualized (200× magnification). The number of migrated cells per field was quantified for **(G)** GHTFs and **(H)** AF. Data are presented as means ± SD. Different lowercase letters are significantly different from each other, as determined by one-way ANOVA with post hoc Duncan test (*P*<0.05).

**Figure 6:**
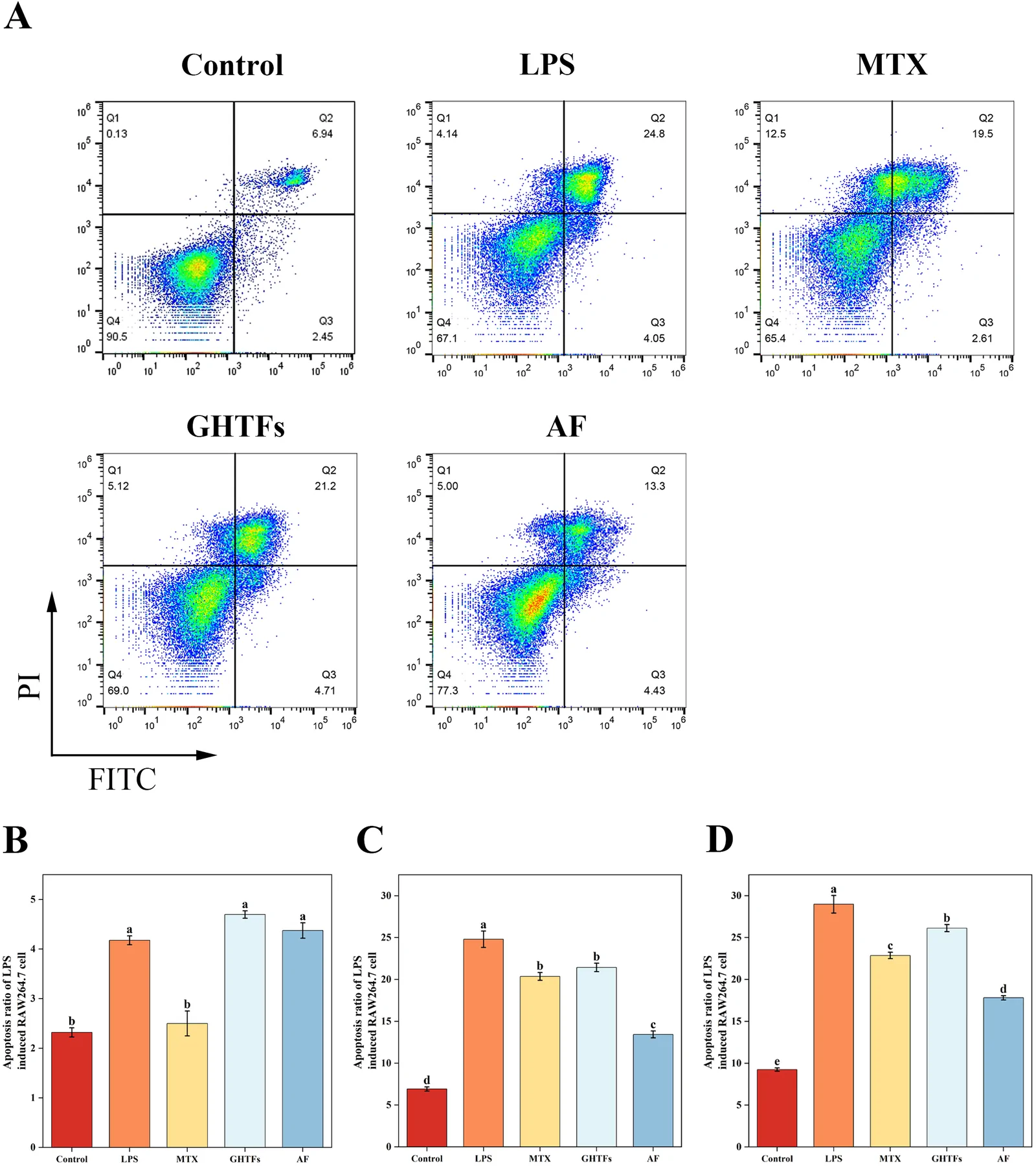
Effects of GHTFs/AF on the apoptosis of RAW264.7 cells. **(A)** Representative scatter plots of the results of the Annexin-V/PI analysis of RAW264.7 cells. Percentages of early apoptotic cells **(B)**, late apoptotic cells **(C)** and total apoptotic cells **(D)** among the RAW264.7 cells in each group. Data are presented as means ± SD. Different lowercase letters are significantly different from each other, as determined by one-way ANOVA with post hoc Duncan test (*P*<0.05).

GHTFs and AF were assessed for their effects on RAW264.7 cell viability (**Supplementary Figures 2A, B**). Both compounds showed no cytotoxicity at concentrations below 125 μg/mL, while concentrations ≥ 31.25 μg/mL inhibited LPS-induced proliferation (**Supplementary Figures 2C, D**). At 125 μg/mL, GHTFs and AF exhibited certain inhibitory activities of 40.75 ± 3.04% and 36.76 ± 3.02%, respectively. Subsequent experiments used 125, 62.5, and 31.25 μg/mL HD (MD, LD). During inflammatory responses, NO acts as a proinflammatory mediator that upregulates inflammatory factor expression in immune cells, particularly macrophages and neutrophils, thereby inducing oxidative stress and tissue damage. The detection of NO content in the cell culture supernatant revealed that LPS stimulation significantly increased the release of NO to a level three times greater than that in the control group (*P* < 0.05), which was attenuated by AF (44–58%) and GHTFs (44–80%) (**Supplementary Figure 3**), with AF showing greater inhibition. Both compounds suppressed LPS-induced macrophage activation, although AF was more effective. Inflammatory cell migration is a central part of the inflammatory response, and its study is important for understanding the occurrence, development, and regulation of inflammation. Wound-healing assays revealed concentration-dependent suppression, with GHTFs reducing healing rates by 16.93–30.82% (24 h) and 24.19–31.94% (48 h), whereas AF showed greater inhibition (24 h: 35.12–39.26%; 48 h: 38.32–44.21%) (**Figures 5A–E**). This result was further confirmed by Transwell Boyden chamber assays (**Figures 5F–H**). The cells stimulated with LPS crossed the membrane successfully, but AF significantly limited transmigration to 6.07–21.4% of that in the LPS group (*P* < 0.05). GHTFs also significantly inhibited invasion, significantly reducing the number of cells that crossed the membrane to 7.34–37.8% of that in the LPS group (*P* < 0.05). Additionally, both compounds reduced LPS-induced apoptosis, although AF exhibited superior efficacy, particularly in late apoptosis (**Figures 6A–D**). Overall, compared with GHTFs, AF consistently demonstrated greater antimigratory and antiapoptotic effects.

Finally, transcriptomic analysis was used to explore the regulatory effects of GHTFS and AF on LPS-induced RAW264.7 cells (**Supplementary Figures 4A–C**). Compared with the LPS controls, the AF treatment resulted in more upregulated genes than downregulated genes, whereas the GHTFs presented balanced differential expression. GO enrichment analysis comparing control and LPS-treated cells highlighted biological processes dominated by stress, cytokine, and defense responses. The extracellular space/region was the prominent cellular component, and cytokine-related activities prevailed in molecular functions, which is consistent with ML predictions. AF predominantly modulated immune processes (BP), cytoplasmic components (CC), and protein binding (MF), whereas GHTFs influenced biological regulation, cell surface components, and receptor binding (**Supplementary Figures 4D–F**). KEGG pathway analysis revealed that the pathways most important for the control group compared with the LPS group were the TNF signaling pathway (*P* = 9.08e^-12^), the IL-17 signaling pathway (*P* = 4.10e^-10^), and cytokine–cytokine receptor interactions (*P* = 5.83e^-10^), which was consistent with the predictions of ML. AF treatment notably affected the PD-1/PD-L1 checkpoint, actin cytoskeleton, and atherosclerosis pathways, whereas GHTFs primarily regulated ferroptosis (*P* = 1.4×10⁻^5^) and mineral absorption (*P* = 4.84×10⁻^5^) (**Supplementary Figures 4G–I**).

### 3.3 GHTFs alleviated the pathology of CIA mice

**Figure 7:**
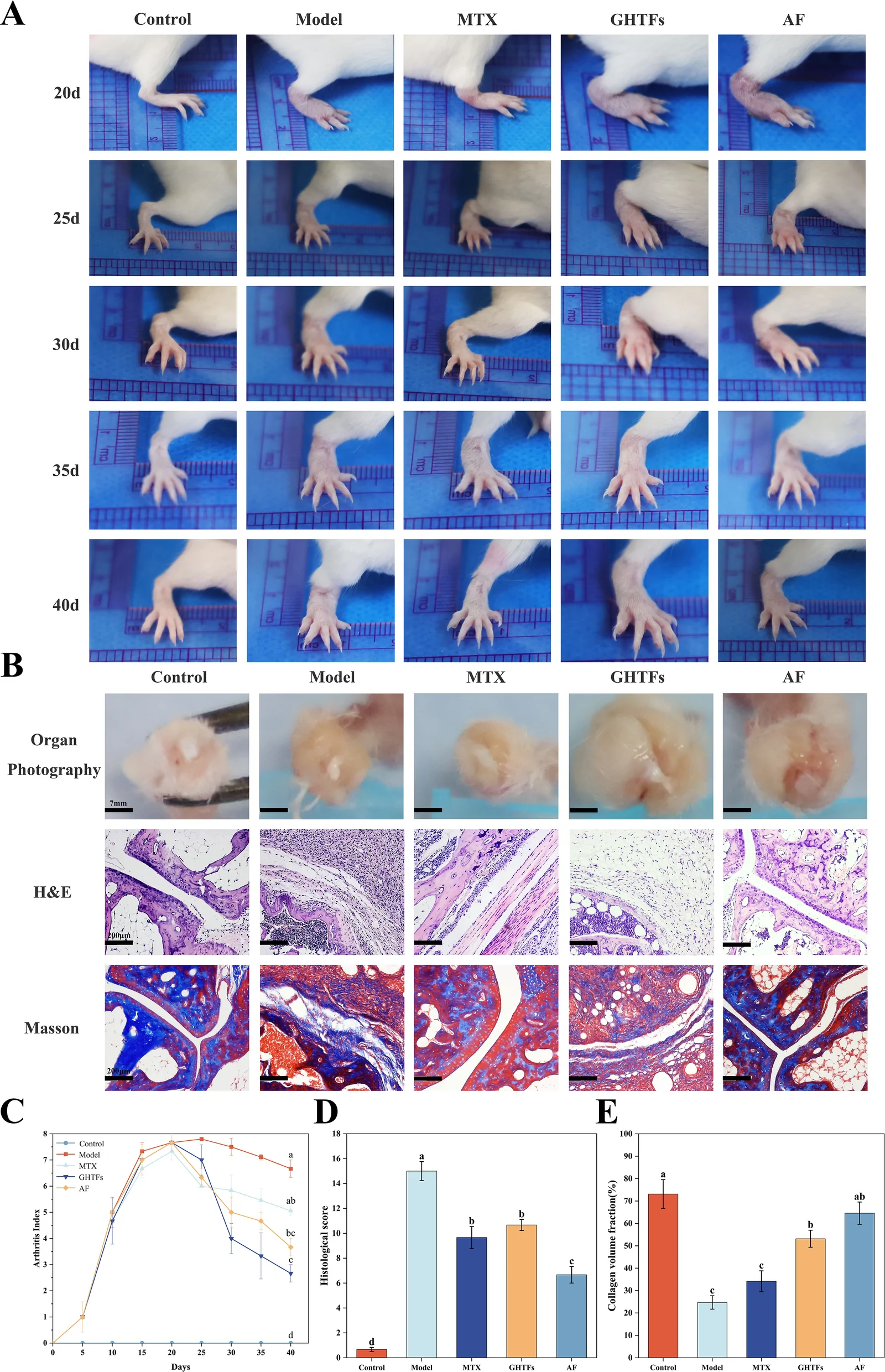
GHTFs treatment ameliorated CIA in mice. **(A)** Representative images of hind paws from the different groups. **(B)** The synovium of the knee joint was sectioned, hematoxylin–eosin-stained and Masson-stained. The scales are 7 mm and 200 μm. **(C)** The clinical scores of the different groups. **(D)** Histological scores of the different groups. **(E)** Masson staining scores of the different groups. Data are presented as means ± SD. Different lowercase letters are significantly different from each other, as determined by one-way ANOVA with post hoc Duncan test (*P*<0.05).

CIA model mice were treated for 20 days (**Figure 7**). To evaluate the effectiveness of GHTFs in CIA mice, clinical scores, histological assessments, and Masson staining results were used as evaluation metrics. Compared with the normal group, the CIA group presented significantly greater foot swelling and RA clinical scores, with the most severe condition observed on day 20 (**Figures 7A, C**). By day 40, the MTX, AF, and GHTFs treatment groups presented marked reductions in foot swelling and RA clinical scores compared with those of the CIA group. Notably, clinical scores were reduced by at least 40% in the GHTFs group compared with those in the CIA group, which was significantly lower than those in the AF and MTX groups, suggesting that GHTFs are superior to AF and MTX in terms of therapeutic efficacy.

The CIA model group exhibited synovial tissue proliferation, articular surface irregularities, vascular opacity formation, and substantial destruction of both cartilage and subchondral bone. Conversely, compared with the model group, the MTX-, AF-, and GHTFs-treated CIA groups presented significant reductions in synovial hyperplasia, inflammatory cell infiltration, and vascular opacity formation (**Figures 7B, D**). The H&E tissue scoring results revealed that the AF group achieved the most pronounced decrease in scores relative to the model group, followed by the MTX and GHTFs groups. A reduction in collagen fibers is one of the most important histopathological features of arthritis. Compared with those in the normal group, Masson’s trichrome staining revealed destruction of joint collagen tissue and severe breakdown and deposition between joints in the CIA model group. The area occupied by collagen fibers was expressed as the collagen volume fraction (CFV). Compared with that of the normal group, the CFV of the model group was reduced by at least 49%. The MTX, GHTFs, and AF groups presented increased CVF. Notably, the CFV of the AF group was 64.56%. These findings indicated that the recovery of the joint collagenous tissues in the AF group was superior to that in the MTX and GHTFs groups. Compared with both the MTX and GHTFs groups, the AF group demonstrated superior recovery of joint collagen tissue (**Figures 7B, E**).

### 3.4 GHTFs modulate the gut microbiota in CIA mice

**Figure 8:**
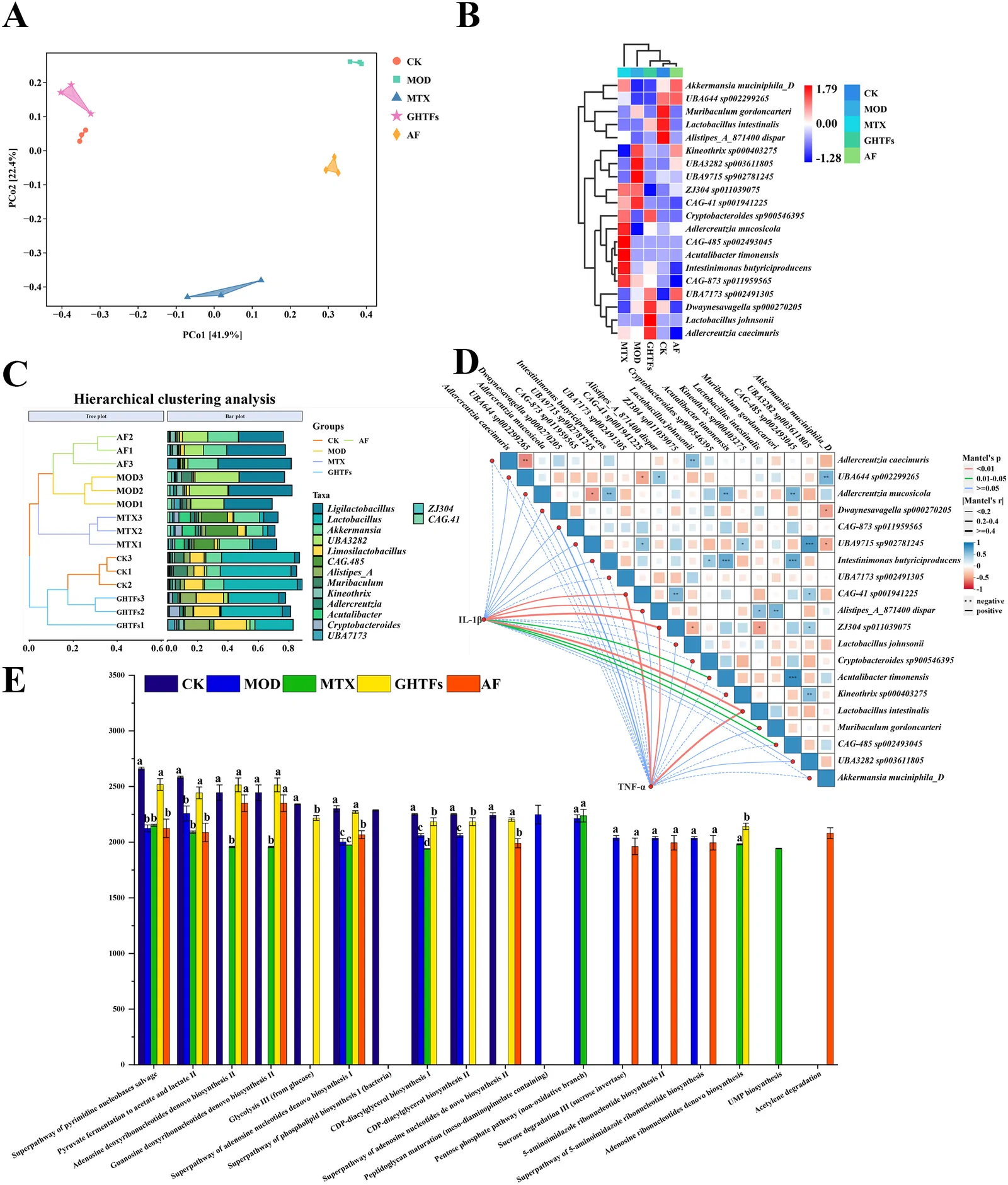
GHTFs regulate the intestinal flora of CIA mice. **(a)** PCoA. **(b)** The left panel is a hierarchical clustering tree plot; the right panel is a stacked histogram of the top 15 genera in terms of abundance. **(c)** Clustered heat diagram of the species composition of the different groups. **(d)** Correlation analysis between the gut microbiota composition at the species level and proinflammatory factors. *p<0.05, **p<0.01, ***p<0.001. **(e)** Functional prediction chart. Data are presented as means ± SD. Different lowercase letters are significantly different from each other, as determined by one-way ANOVA with post hoc Duncan test (*P*<0.05).

To understand the effects of GHTFs on arthritis-induced dysbiosis, the microbial diversity of feces was analyzed. PCA revealed substantial variations in β diversity among the five study groups (**Figure 6A**). Hierarchical clustering analysis revealed highly similar compositions of the intestinal flora in CIA mice treated with GHTFs compared with those in control mice, with bacteria from the genera *Limosilactobacillus* (CIA: 0.96%, GHTFs: 18.77%), *Lactobacillus* (CIA: 0.26%, GHTFs: 32.81%), and others predominant. However, the intestinal flora of the CIA model group was similar to that of the AF-treated group, with bacteria predominantly from the genera *Ligilactobacillus* (CIA: 34.33%, AF: 38.68%) (**Figures 8A, B**). Heatmap analysis revealed that the main strains in the control group were *Muribaculum gordoncarteri* (6.13%), *Lactobacillus intestinalis* (7.28%), and *Akkermansia muciniphila_D* (10.69%), whereas the main strains in the CIA mouse model group treated with GHTFs were *Lactobacillus johnsonii* (6.04%), *Cryptobacteroides* sp*900546395* (3.37%), and *Dwaynesavagella* sp*000270205* (1.35%). Notably, *L. johnsonii* (CK: 0.13%, CIA: 0.00%, GHTFs: 6.04%) and *L. intestinalis* (CK: 7.28%, CIA: 0.03%, GHTFs: 3.55%) belong to the same genus. However, the strains in the CIA mouse model group were mainly *UBA3282* sp*003611805* (25.30%), *UBA9715* sp*902781245* (2.96%), and *CAG-41* sp*001941225* (2.53%) (**Figure 8C**). To validate the correlation between proinflammatory cytokines and microbial communities, we correlated the composition of taxonomic strains with the composition of inflammatory cytokines at the species level. The results demonstrated that *ZJ304* sp*011039075*, *Alistipes_A_871400 dispar*, *CAG-41* sp*001941225*, *UBA7173* sp*002491305*, *CAG-485* sp*002493045*, *L. intestinalis*, and *Kineothrix* sp*000403275* were positively correlated with TNF-α and IL-1β (*P* < 0.05). However, *Acutalibacter timonensis*, *Cryptobacteroides* sp. *900546395*, and *Muribaculum gordoncarteri* were positively correlated with only IL-1β (*P* < 0.05) (**Figure 8D**). Notably, *L. intestinalis* and *L. johnsonii* in control and GHTFs-treated CIA mice were negatively correlated with strains *UBA9715* sp*902781245*, *CAG-41* sp*001941225*, *Kineothrix* sp*000403275*, and *UBA3282* sp*003611805* in CIA mice (*P* < 0.05) (**Figures 8C, D**). Using PICRUST software for functional prediction of the intestinal microbiota, we observed that multiple functions were markedly diminished in the CIA mouse model group, the MTX group, and the AF group relative to the normal group. In contrast, these functions were restored to normal levels in the GHTFs-treated group (**Figure 8E**).

GHTFs uniquely restored gut microbiota homeostasis in CIA mice by enriching beneficial *Lactobacillus* spp., such as *L. johnsonii*, *L. intestinalis*, and *Limosilactobacillus*, while decreasing the enrichment of the unclassified proinflammatory strain *UBA3282*. A negative correlation was observed between *Lactobacillus* spp. and the inflammatory markers TNF-α and IL-1β. Furthermore, compared with the AF and MTX treatments, the GHTFs resulted in superior microbial metabolic recovery.

### 3.5 GHTFs mitigate CIA in mice *via* immune modulation

To understand the immunomodulatory effects of GHTFs in CIA mice, immunohistochemical and ELISA analyses were performed. First, we performed immunohistochemical analyses of MMP3 and MMP9, the two targets with the strongest binding energy to AF according to the molecular docking results. Compared with those in the CIA model group, the expression of MMP3 and MMP9 target proteins in the MTX, GHTFs, and AF treatment groups decreased significantly, and the expression of MMP3 and MMP9 target proteins in the GHTFs treatment group was significantly lower than that in the MTX and AF groups (**Supplementary Figures 5A, B**). Moreover, it was observed that the concentrations of proinflammatory cytokines, TNF-α and IL-1β, were significantly reduced in the MTX, GHTFs, and AF groups compared to the CIA model group. Moreover, the concentrations of TNF-α and IL-1β were notably lower in the GHTFs-treated group than in the MTX- and AF-treated groups (**Supplementary Figures 5C, D**). In addition, WB analysis of the target proteins ERK2, NF-κB, and NLRP3, which are essential for inflammation-related signaling pathways in synovial tissues, revealed significantly lower protein expression in the MTX-, GHTFs-, and AF-treated groups than in the CIA mouse model group. Notably, ERK2 and NF-κB expression in the GHTFs-treated group was comparable to that in the normal group, whereas the expression of NLRP3 was markedly lower than that in all other groups (**Supplementary Figure 6**).

## 4 Discussion

RA is a common autoimmune disease, and increasing evidence suggests that alterations in the gut flora precede its onset, demonstrating a significant association between the gut microbiota and the occurrence of RA (51). *G. hypoleucum* belongs to the genus Gnaphalium in the family compositae, and its total flavonoid content is very high (10). Previous studies have shown that total flavonoids can regulate the intestinal flora to increase immunity and have antioxidant, anti-inflammatory, and antimicrobial effects (6–8, 11, 14). In this study, we systematically evaluated the therapeutic effects of GHTFs in CIA mice and demonstrated their ability to ameliorate RA symptoms through gut microbiota modulation and immune regulation. GHTFs significantly ameliorated CIA in mice by reducing joint swelling (*P* < 0.05) and suppressing proinflammatory cytokines (TNF-α and IL-1β) and key mediators (MMP3/9, ERK2, NF-κB, and NLRP3). Importantly, GHTFs reshaped the gut microbiota in CIA mice by enriching beneficial bacterial species such as *L. johnsonii* and *L. intestinalis*, suggesting a link between microbial regulation and RA alleviation. ML (AUC = 0.959) and SHAP analysis identified EGFR, VEGFA, MMP13, POLB, and MMNK2 as key targets, with experimental validation confirming the antiarthritic effects of AF, demonstrating the therapeutic potential of GHTFs via gut–joint axis regulation.

The apparent discrepancy between ML-predicted key targets (EGFR, VEGFA, MMP13, POLB, and MMNK2) and our final focus on MMP9 stems from the subsequent validation steps. While these predicted targets showed strong associations in our ensemble model (AUC = 0.959), molecular docking and dynamics simulations revealed that AF exhibited superior binding affinity and stability with MMP9 (ΔG = -10.5 kcal/mol, RMSD < 1.8 Å), establishing it as the most pharmacologically relevant target. In RA, MMP3 and MMP9 promote the migration of immune cells, synoviocytes, and endothelial cells through the degradation of extracellular matrix (ECM) components, thereby exacerbating the inflammatory response and destruction of the joints. MMP3 promotes the proliferation and migration of synoviocytes through the degradation of collagen and the enhancement of ECM remodeling, resulting in the destruction of articular cartilage, whereas MMP9 helps immune cells cross the vessel wall into the joint by degrading gelatin and collagen, enhancing the inflammatory response and further aggravating joint damage (52). This conclusion was subsequently verified by experiments in which AF was more effective than GHTFs in inhibiting the proliferation and migration of inflammatory cells in cellular experiments, and the positive cell areas in the AF group were indeed slightly smaller than those in the GHTFs group according to the immunohistochemical results. Interestingly, the therapeutic effects of AF differ from those of the cellular and CIA mouse models on the basis of the active ingredients in GHTFs identified through screening. While the single-target action of AF on MMP9 explains its excellent *in vitro* performance, the broader therapeutic effects of GHTFs *in vivo* may stem from their multitarget ability to simultaneously modulate the immune response and inhibit the expression of ERK, NF-kB, and NLRP3 proteins, thereby decreasing the levels of the inflammatory factors TNF-α and IL-1β and increasing the colonization of the beneficial intestinal bacteria *Lactobacillus* spp. Reducing the enrichment of intestinal proinflammatory bacteria spp. restores homeostasis of the intestinal microbiota, which ultimately leads to better therapeutic efficacy of GHTFs than AF in CIA animal models. This suggests that complex botanical formulations can surpass the therapeutic effects of single compounds in a holistic organismal setting through systems-level modulation (53, 54). Motahar et al. conducted a clinical trial comparing the therapeutic effects of turmeric extract and naproxen in patients with RA across different disease stages. These findings revealed that the whole turmeric extract exhibited superior efficacy compared with its individual monomeric constituents, which aligns with the results observed in our study (55).

AF, luteolin, and apigenin are hypothesized to be the active ingredients of GHTFs, contributing to their therapeutic effects in RA. Previous studies have demonstrated that AF can reduce the release of inflammatory cytokines by inhibiting endotoxin-induced activation of Kupffer cells, which in turn inhibits the activation of the downstream nuclear factor NF-κB (56). Luteolin has anti-inflammatory effects and can attenuate the inflammatory response by downregulating the TLR/MyD88/NF-κB pathway and by inhibiting the expression of a number of inflammatory factors, such as IL-1β, IL-6, IL-17, and TNF-α (57). The NLRP3 inflammasome, a multiprotein complex encompassing cysteine protease-1, regulates the secretion of IL-1β. This inflammasome is imperative for the innate immune response. Luteolin has been shown to inhibit the activation of the NLRP3 inflammasome by obstructing ASC oligomerization, thereby significantly diminishing its expression (58). Apigenin inhibits the activation of NF-κB and MAPKs and reduces the production of cytokines such as IL-1β, IL-6, and TNF-α, thus suppressing the inflammatory response (59). Additionally, we identified MMP9 and MMP3 as potential therapeutic targets of GHFTs in the treatment of RA, given their pivotal role in the pathogenic processes of this disease. In patients with RA, cytokines such as IL-6 and TNF-α can be directly involved in the tissue inflammatory response and contribute to the upregulation of the levels of MMPs in the blood and synovial fluid (50, 60). Previous studies have shown that a variety of MMPs, such as MMP1, MMP3, and MMP9, are secreted in response to cytokines and that their expression is upregulated in synovial fibroblasts, which are thought to play a key role in the degeneration of articular cartilage and joint damage in RA patients (61). In addition, RNA sequencing analysis of synovial biopsies has shown that IL-8 and TOP2A are pivotal proteins in the regulation of oxidative phosphorylation and Toll-like receptor signalling pathways. These pathways have been proven to play an instrumental role in the development of RA (62).

The findings from the KEGG pathway analysis revealed that GHTFs exert their therapeutic effects on RA by modulating signaling pathways, including the IL-17 signaling, TNF signaling, and T-cell receptor signaling pathways. Notably, GHTFs also influence the RA-specific signaling pathway. IL-17 is a proinflammatory cytokine that attracts neutrophils to the site of reaction to promote more inflammatory reactions and autoimmune diseases, as well as stimulate the release of more IL-6, TNF-α, and IL-1β from chondrocytes, synoviocytes, macrophages, and osteoblasts (63). Moreover, IL-17 synergistically enhances the inflammatory response alongside cytokines such as IL-6 and TNF-α, recruiting additional inflammatory cells to the inflammation site and exacerbating joint damage (64). This study demonstrated that GHTFs are capable of reducing the serum concentrations of cytokines such as IL-1β and TNF-α. Therefore, GHTFs may treat RA by modulating the IL-17 signaling pathway. Furthermore, in RA, signaling occurs mainly through the NF-κB pathway. TNF-α is involved in positive feedback regulation in the TNF signaling pathway, and the activation of NF-κB is one of the most important downstream events (65). Activation of the NF-κB signaling pathway can further regulate cytokines, such as MMP1, MMP3, and IL-6, to promote inflammatory responses (66).

Changes in the gut microbiota are essential for the pathogenesis of RA (67). Our results showed that GHTFs alleviate RA symptoms by improving the intestinal flora and modulating the organism’s immunity. We detected high abundances of strains of the genera *Lactobacillus* and *Limosilactobacillus* through hierarchical clustering analysis in both control and GHTFs-treated CIA mice. However, the abundances of these two strains were lower in the CIA mouse model group and in the AF- and MTX-treated CIA mice. *Lactobacillus* and *Limosilactobacilli* play crucial roles in preventing pathogen invasion and maintaining intestinal function (68, 69). In addition, the oral ingestion of *Lactobacillus* has been demonstrated to attenuate the progression of RA and reduce inflammation in animal models of CIA (70–72). Heatmap analysis of the species composition revealed that *L. johnsonii* and *L. intestinalis*, which belong to the genus *Lactobacillus*, can modulate the interaction between the intestinal epithelium and the immune system, with potential therapeutic effects to inhibit RA (73–76). Correlation analysis of the gut microbiota composition with proinflammatory factors revealed that *L. johnsonii* and *L. intestinalis* were negatively correlated with *UBA3282* sp*003611805*, *UBA9715* sp*902781245*, and *CAG-41* sp*001941225* in CIA mice and positively correlated with TNF-α and IL-1β. It is hypothesized that large amounts of TNF-α and IL-1β are produced in RA, while *L. johnsonii* and *L. intestinalis* proliferate to regulate the interaction between intestinal epithelial cells and the immune system. Moreover, *L. johnsonii* and *L. intestinalis* and their metabolites might alleviate the symptoms of RA by inhibiting the proliferation of *UBA3282* sp*003611805*, *UBA9715* sp*902781245*, and *CAG-41* sp*001941225*. However, more experiments are needed to further validate this finding.

The predictions made by the PICRUST software programme indicated a decline in the abundance of intestinal flora associated with metabolic pathways involving sugar, lipids and proteins in CIA mice, whereas the abundance of sugar, lipid, and protein metabolic pathways was restored to normal levels in the GHTFs-treated group. The metabolism of sugars, lipids, and proteins is closely related to RA (77). Short-chain fatty acids are capable of modulating B-cell and T-cell differentiation, thereby diminishing RA autoantibody generation, whereas tryptophan, an aromatic amino acid, suppresses macrophage-mediated proinflammatory responses and enhances intestinal barrier function. (78–80). GHTFs are speculated to ameliorate RA by modulating the metabolites of the intestinal flora.

While this study demonstrates the promising therapeutic potential of GHTFs in RA, several limitations should be acknowledged. Firstly, our findings are based primarily on a CIA model and RAW264.7 macrophage cell lines, which, although widely used, may not fully capture the complexity and heterogeneity of human RA. Secondly, although GHTFs have shown the ability to modulate the gut microbiota by enriching beneficial Lactobacillus species, a direct causal relationship between these microbial alterations and RA improvement remains to be confirmed. Future studies should consider employing fecal microbiota transfer (FMT) experiments to establish causality. Furthermore, gnotobiotic models could be utilized to explore the detailed interplay between the microbiota and immune system, providing deeper insights into the mechanistic pathways involved. Lastly, pharmacokinetic profiling and safety assessments are crucial for assessing the translational potential of GHTFs as a multi-target therapeutic strategy for RA.

## 5 Conclusion

This study revealed that GHTFs have superior therapeutic effects against rheumatoid arthritis by combining potent direct anti-inflammatory activity with immune modulation and gut microbiota restoration. AF, as the key component, primarily drives anti-inflammatory responses, whereas GHTFs synergistically reshape systemic immunity and microbial ecology to achieve enhanced disease amelioration. These findings position GHTFs as a promising multitarget strategy with strong translational potential for RA treatment.

## Data Availability

The raw data supporting the conclusions of this article will be made available by the authors, without undue reservation.

## Glossary

AF: Amentoflavone
BP: Biological process
CC: Cell Component
CFV: Collagen volume fraction
CIA: Collagen induced arthritis
ECM: Cell-extracellular matrix
EGFR: Epidermal growth factor receptor
ELISA: Enzyme-Linked Immunosorbent Assay
Enet: Elastic net
FBS: Fetal bovine serum
FMT: Fecal microbiota transfer
GBM: Gradient boosting machine
*G. hypoleucum* DC: *Gnaphalium hypoleucum* DC.
GHTFs: Total flavonoids of *G. hypoleucum* DC
GO: Gene ontology
HRMS: High resolution mass spectrometry
IL-17: Interleukin-17
KEGG: Kyoto encyclopedia of genes and genomes
LC–MS: Liquid chromatography-Mass spectrometry
LDA: Linear discriminant analysis
LPS: Lipopolysaccharide
MAPK: Mitogen-Activated protein kinase
MF: Molecular function
MMPs: Matrix metalloproteinases
MMP13: Matrix Metalloproteinase 13
ML: Machine learning
MS: Mass spectrometry
MTX: Methotrexate
NLRP3: NOD-like receptor family pyrin domain containing 3
PCA: Principal component analysis
PI3K–Akt: Phosphoinositide 3-kinase signaling pathway
RA: Rheumatoid arthritis
RF: Random forest
RPMI-1640: Roswell park memorial institute 1640
SHAP: SHapley Additive exPlanations
SVA: Surrogate variable analysis
SVM: Support vector machine
Th17: T helper cell 17
UPLC: Ultra performance liquid chromatography
VEGFA: Vascular endothelial growth factor A.
