# Correction: Machine learning reveals targets of *Gnaphalium hypoleucum* DC. flavonoids against rheumatoid arthritis through gut microbiota and anti-inflammation

**DOI:** 10.3389/fimmu.2026.1954690

**Published:** 2026-09-15

**Authors:** Yu-Long Li, Zi-Yong Chu, Ding-Hui Xu, Shu-Yun Wei, Ya-Si Nong, Xiao-Xi Luo, Yi-Jing Wang, Hong Zeng

**Affiliations:** 1 Technology Innovation Cooperation Base of Prevention and Control Pathogenic Microbes with Drug Resistance, School of Basic Medicine, Youjiang Medical University for Nationalities, Baise, China; 2 College of Life Science and Technology, Xinjiang University, Urumqi, China; 3 School of Biomedical and Pharmaceutical Sciences, Guangdong University of Technology, Guangzhou, China

**Keywords:** intestinal microflora, machine learning, molecular docking, rheumatoid arthritis (RA), total flavonoids of *G. hypoleucum* DC. (GHTFs)

There was a mistake in **Figure 5** as published. Our experiment generated a substantial number of microscopy images, and because many of them were visually similar, an error occurred in figure assembly: **Figure 5A** “MTX-24h” has a layout mistake in its image arrangement. The corrected **Figure 5** appears below.

**Figure 5:**
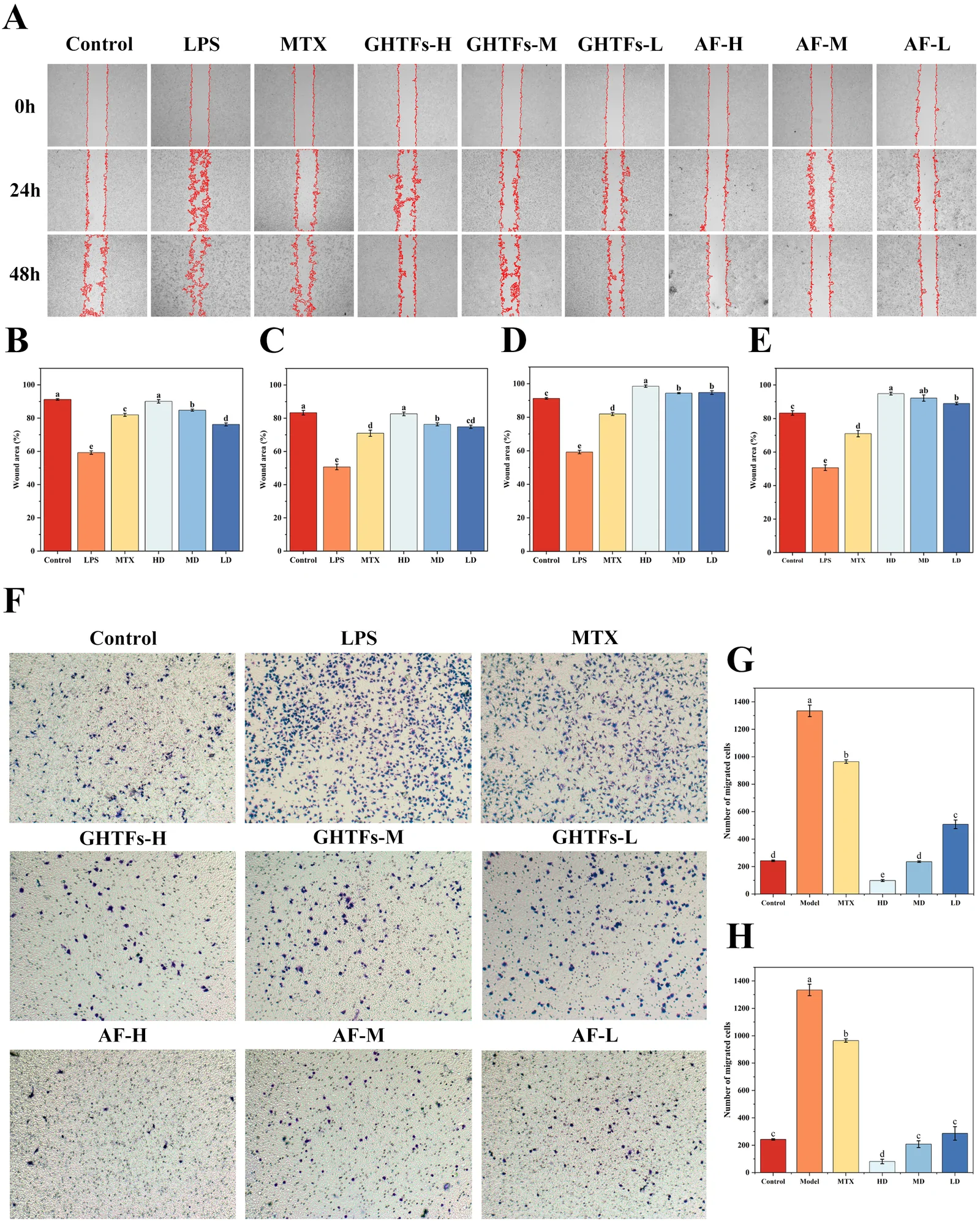
GHTFs/AF suppress the migration of LPS-induced RAW264.7 cells. **(A)** The antimigratory effects of GHTFs and AF (125, 62.5, and 31.25 μg/mL) were assessed via a scratch wound-healing assay. Wound closure was monitored at 0, 24, and 48 h under a microscope (100× magnification). Migration inhibition was quantified by measuring the percentage reduction in the scratch width compared with the initial wound area. Representative images illustrate the effects of **(B)** 24 h of treatment with GHTFs, **(C)** 48 h of treatment with GHTFs, **(D)** 24 h of treatment with AF, and **(E)** 48 h of treatment with AF. The data are presented as the means ± SEMs from at least three independent experiments. **(F)** Further validation was performed via a Transwell migration assay after 24 h of treatment. The migrated cells were fixed, stained, and visualized (200× magnification). The number of migrated cells per field was quantified for **(G)** GHTFs and **(H)** AF. Data are presented as means ± SD. Different lowercase letters are significantly different from each other, as determined by one-way ANOVA with post hoc Duncan test (P<0.05).

The original version of this article has been updated.

