# Supplementary material for: Machine learning reveals targets of Gnaphalium hypoleucum DC. flavonoids against rheumatoid arthritis through gut microbiota and anti-inflammation
Source: Front Immunol. 2026 Mar 6;17:1732859. doi: 10.3389/fimmu.2026.1732859 (PMC13002369; doi:10.3389/fimmu.2026.1732859)
Supplement: Supplementary file 1 [file DataSheet1.doc]

**Machine learning guided discovery of *Gnaphalium hypoleucum* DC. Flavonoids as multi-target Rheumatoid Arthritis therapeutics via anti-inflammatory and gut microbiota regulation**

**Supplementary Materials**

**The file includes**

Materials and Methods

Fig. S1 Molecular Docking Results

Fig. S2 GHTFs and AF on the cell viability and proliferation of RAW264.7 cells

Fig. S3 Detection of NO content

Fig. S4 Transcriptomics analysis

Fig. S5 The immunohistochemical and enzyme-linked immunosorbent assay

Fig. S6 The western blot analysis of synovial joint tissues

Table S1 Flavonoids information table

Table S2 Arthritis scoring criteria

**Materials and methods**

**Reagents and Materials**

The Amentoflavone (AF, CAS registry No. 1617-53-4) was purchased from Chengdu Herbpurify CO., LTD (Chengdu, China). NLRP3 Rabbit pAb (A21906, ABclonal, USA), β-Actin Rabbit pAb (AC038, ABclonal, USA), ERK2 Rabbit pAb (A0229, ABclonal, USA), NF-κB P65/RelA Rabbit pAb (A2547, ABclonal, USA).

**Analysis of differentially expressed genes**

Transcriptomic data were analyzed using the limma package, and differentially expressed genes (DEGs) were defined as those with an FDR-adjusted P value < 0.05 and an absolute log₂ fold change greater than 0.585 (equivalent to a 1.5-fold change). Data visualization was performed using the ggplot2 package (1).

**Weighted gene co-expression network analysis**

A scale-free gene co-expression network was constructed using the WGCNA package(2). Sample quality control was performed by hierarchical clustering to remove outlier samples. Soft-thresholding power was determined using the scale-free topology criterion (scale-free R² > 0.85). Module detection was conducted by hierarchical clustering of the topological overlap matrix (TOM), with parameters set to minModuleSize = 30 and mergeCutHeight = 0.25. Module–trait associations were assessed by correlating module eigengenes with phenotypic traits using Pearson correlation (|r| > 0.5, P < 0.05). Finally, hub genes within each module were identified based on high intramodular connectivity (kME ≥ 0.8)(3).

**Identification of therapeutic targets associated with RA**

To identify potential therapeutic targets for RA, a cross-analysis was performed among differentially expressed genes (DEGs), WGCNA module genes, and GHFs-predicted targets. The overlapping genes were visualized using a Venn diagram. Subsequently, the therapeutic target network associated with pyocyanin-exacerbated sepsis was constructed and analyzed using the STRING database (version 12.0, https://cn.string-db.org/) and visualized with Cytoscape (version 3.10.3)(4,5).

**Functional enrichment analysis**

The clusterProfiler package was used to perform Gene Ontology (GO) analysis—covering biological process (BP), cellular component (CC), and molecular function (MF)—and Kyoto Encyclopedia of Genes and Genomes (KEGG) pathway analysis (P < 0.05). These analyses elucidated the mechanisms through which pyocyanin exacerbates sepsis(6). Gene Set Enrichment Analysis (GSEA) was conducted using the official c5.go.symbols.gmt and c2.cp.kegg.Hs.symbols.gmt gene sets to explore the significantly enriched biological pathways and functional categories associated with the core therapeutic targets for RA treatment with GHFs (7). A significance threshold of P < 0.05 was applied.

**SHAP-based interpretation of model predictions**

To interpret the predictions of machine learning models, we applied the SHAP (SHapley Additive exPlanations) algorithm, which quantifies the contribution of each feature to model outputs(8). A series of SHAP visualizations—including cumulative contribution curves, summary plots, beeswarm plots, scatter plots, and force plots—were generated to illustrate both global and sample-specific feature importance.

**Cell viability assay**

Cell Count Kit-8 (BMC, China) was used to determine the effect of GHTFs and AF on cell viability. Cells (5 × 10^4^ cells/well) (9) were digested by 0.25% trypsin (Solarbio, China), and then added into 96-well plates. After culturing for 24 h, cells were incubated with different concentrations of GHTFs and AF (0-1000 μg/mL) for 24 h, and then 10 μL CCK8 solution was added to each well. The cells were incubated at 37 °C for 3 h, and then the supernatant was used to measure absorbance at 450 nm wavelength using a photometer.

**Cell proliferation assay**

Cell proliferation assay used to estimate the effects of GHTFs and AF on the proliferation of LPS induced RAW264.7 cells. In brief, RAW264.7 cells were cultured with LPS (100 ng/ml, final concentration in the well) for 24 h, followed by incubating with GHTFs and AF (0-1000 μg/mL) (10). At the end of treatment, the 10 μL CCK8 solution was added to each well. The cells were incubated at 37 °C for 3 h, and then the supernatant was used to measure absorbance at 450 nm wavelength using a photometer, and the cell inhibition rate (%) was calculated.

**Measurement of NO on RAW 264.7**

Add 200 µL of cells at a concentration of 5 × 10⁵ to each well of a 96-well plate, discard the culture medium, wash once with PBS, and then add the medium containing different concentrations of GHTFs or AF. After 48 h of treatment, 50 μL of supernatant, 50 μL of Giess1 and 50 μL of Giess2 were taken and the absorbance was measured at OD_540_.

**Transcriptomics analysis**

Total RNA was extracted using Trizol reagent (Invitrogen), and its quality and integrity were assessed with a Nano Drop spectrophotometer (Thermo Scientific) and Bioanalyzer 2100 system (Agilent). RNA-seq was conducted on the Illumina platform by Nanjing Personalbio Gene Technology Co., Ltd (Nanjing, China). Gene Ontology (GO) and KEGG pathway enrichment analyses were performed for differentially expressed genes. Results were generated using the Personalbio genes cloud platform (https://www.genescloud.cn/).

**Clinical scoring**

Macroscopic scoring of mouse paws was performed every 5 d during the experiment to assess the progression of CIA. At the same time, the severity of arthritis was scored by two researchers in a blinded manner. Each paw was scored and the 4 scores were summed to give a maximum of 16 points per mouse and 4 points per paw **(Table S2)**(9).

**Immunohistochemical of murine ankle joint tissues**

The synovial tissue of the mice was initially fixed using a 4% paraformaldehyde solution and subsequently embedded in paraffin. The tissue was then subjected to a heating process in an oven at 60 °C for a duration of 1 h, followed by dewaxing in xylene. The tissue underwent a series of polarizations in decreasing concentrations of alcohol (100%, 95%, 75%) and was rinsed with water. To inhibit endogenous peroxidase activity, the tissue was incubated with a 3% H_2_O_2_ solution at room temperature. The epitopes were incubated with 20% normal goat serum for 1 h, and then incubated with primary antibody (MMP3, MMP9) at 4 °C for 12 h to block the epitopes. Upon completion of three water washes, the slides were exposed to HRP-labeled secondary antibody and allowed to incubate for 1 h under room temperature conditions (11). Elution of PBS solution, add DAB chromogenic 5-10 min, elution, H&E staining, dehydration, transparency, sealing. The stained area size was measured using Image J software 1.53 t.

**Enzyme linked immunosorbent assay (ELISA)**

After the 20 d of treatment, approximately 0.5 mL of blood samples were collected from each murine eye and allowed to stand at room temperature for 1 h. The serum was isolated through centrifugation at 2,000 g for a duration of 10 min, subsequently stored at -80 °C for future use. The concentrations of IL-1β and TNF-α in the serum were quantified utilizing an ELISA kit (Jiangsu Meiman Industrial Co., Ltd., Jiangsu, China), adhering to the manufacturer’s guidelines.

**Western blotting (WB)**

Western blot analysis was employed to ascertain the expression levels of ERK, NF-κB, NLRP3, and β-actin in the synovial tissue of murine. Protein extraction from the synovial tissue was conducted on the 20 d of treatment using RIPA lysis buffer supplemented with 1% PMSF. The protein concentration was determined utilizing the BCA protein assay kit. Equivalent quantities of protein samples were then subjected to 10% sodium dodecyl sulfate polyacrylamide gel electrophoresis, followed by transfer to polyvinylidene fluoride membranes (MilliporeSigma, U.S. & Canada). Following blocking with Protein Free Rapid Blocking Buffer (1X) (Epizyme, China) for 10 min, the membranes were incubated overnight at 4 °C with the respective antibody. After rinsing with 0.1% Tween-20 in PBS, the membranes were then incubated for 1 h at room temperature with goat anti-rabbit IgG (H & L)-HRP secondary antibody while shaking horizontally. The protein bands were detected using an enhanced chemiluminescence reagent, and the resulting images were analyzed and normalized to β-actin using Image J software. All assays were performed in triplicate for statistical validity (12).

1. Ritchie ME, Phipson B, Wu D, Hu Y, Law CW, Shi W, Smyth GK. limma powers differential expression analyses for RNA-sequencing and microarray studies. *Nucleic Acids Research* (2015) 43:e47–e47. doi: 10.1093/nar/gkv007

2. Langfelder P, Horvath S, Cai C, Dong J, Miller J, Song L, Yip A, Zhang B. WGCNA: Weighted correlation network analysis. (2010) doi: 10.32614/cran.package.wgcna

3. Gao J, Zhang M, Chen Q, Ye K, Wu J, Wang T, Zhang P, Feng G. Integrating machine learning and molecular docking to decipher the molecular network of aflatoxin b1-induced hepatocellular carcinoma. *International Journal of Surgery* (2025) doi: 10.1097/js9.0000000000002455

4. Szklarczyk D, Kirsch R, Koutrouli M, Nastou K, Mehryary F, Hachilif R, Gable AL, Fang T, Doncheva NT, Pyysalo S, et al. The STRING database in 2023: Protein–protein association networks and functional enrichment analyses for any sequenced genome of interest. *Nucleic Acids Research* (2023) 51:D638–D646. doi: 10.1093/nar/gkac1000

5. Otasek D, Morris JH, Bouças J, Pico AR, Demchak B. Cytoscape automation: Empowering workflow-based network analysis. *Genome Biol* (2019) 20:185. doi: 10.1186/s13059-019-1758-4

6. Wu T, Hu E, Xu S, Chen M, Guo P, Dai Z, Feng T, Zhou L, Tang W, Zhan L, et al. clusterProfiler 4.0: A universal enrichment tool for interpreting omics data. *The Innovation* (2021) 2:100141. doi: 10.1016/j.xinn.2021.100141

7. Subramanian A, Tamayo P, Mootha VK, Mukherjee S, Ebert BL, Gillette MA, Paulovich A, Pomeroy SL, Golub TR, Lander ES, et al. Gene set enrichment analysis: A knowledge-based approach for interpreting genome-wide expression profiles. *Proc Natl Acad Sci USA* (2005) 102:15545–15550. doi: 10.1073/pnas.0506580102

8. Qi X, Wang S, Fang C, Jia J, Lin L, Yuan T. Machine learning and SHAP value interpretation for predicting comorbidity of cardiovascular disease and cancer with dietary antioxidants. *Redox Biology* (2025) 79:103470. doi: 10.1016/j.redox.2024.103470

9. Zheng Q, Du L, Zhao Y, Zhang Z, Piao S, Wang Y, Pan Z. Mechanism of *Rhodiola rosea* – *Euonymus alatus* drug pair against rheumatoid arthritis: Network pharmacology and experimental validation. *Immunity Inflam &amp; Disease* (2023) 11:e1127. doi: 10.1002/iid3.1127

10. Zhang M, Ren H, Li K, Xie S, Zhang R, Zhang L, Xia J, Chen X, Li X, Wang J. Therapeutic effect of various ginsenosides on rheumatoid arthritis. *BMC Complement Med Ther* (2021) 21:149. doi: 10.1186/s12906-021-03302-5

11. Ba X, Huang Y, Shen P, Huang Y, Wang H, Han L, Lin WJ, Yan HJ, Xu LJ, Qin K, et al. WTD attenuating rheumatoid arthritis via suppressing angiogenesis and modulating the PI3K/AKT/mTOR/HIF-1α pathway. *Front Pharmacol* (2021) 12:696802. doi: 10.3389/fphar.2021.696802

12. Chu Z-Y, Li Y-L, Wang L, Wei S-Y, Yang S-Q, Zeng H. Perillaldehyde: A promising antibacterial agent for the treatment of pneumonia caused by acinetobacter baumannii infection. *Int Immunopharmacol* (2024) 126:111311. doi: 10.1016/j.intimp.2023.111311


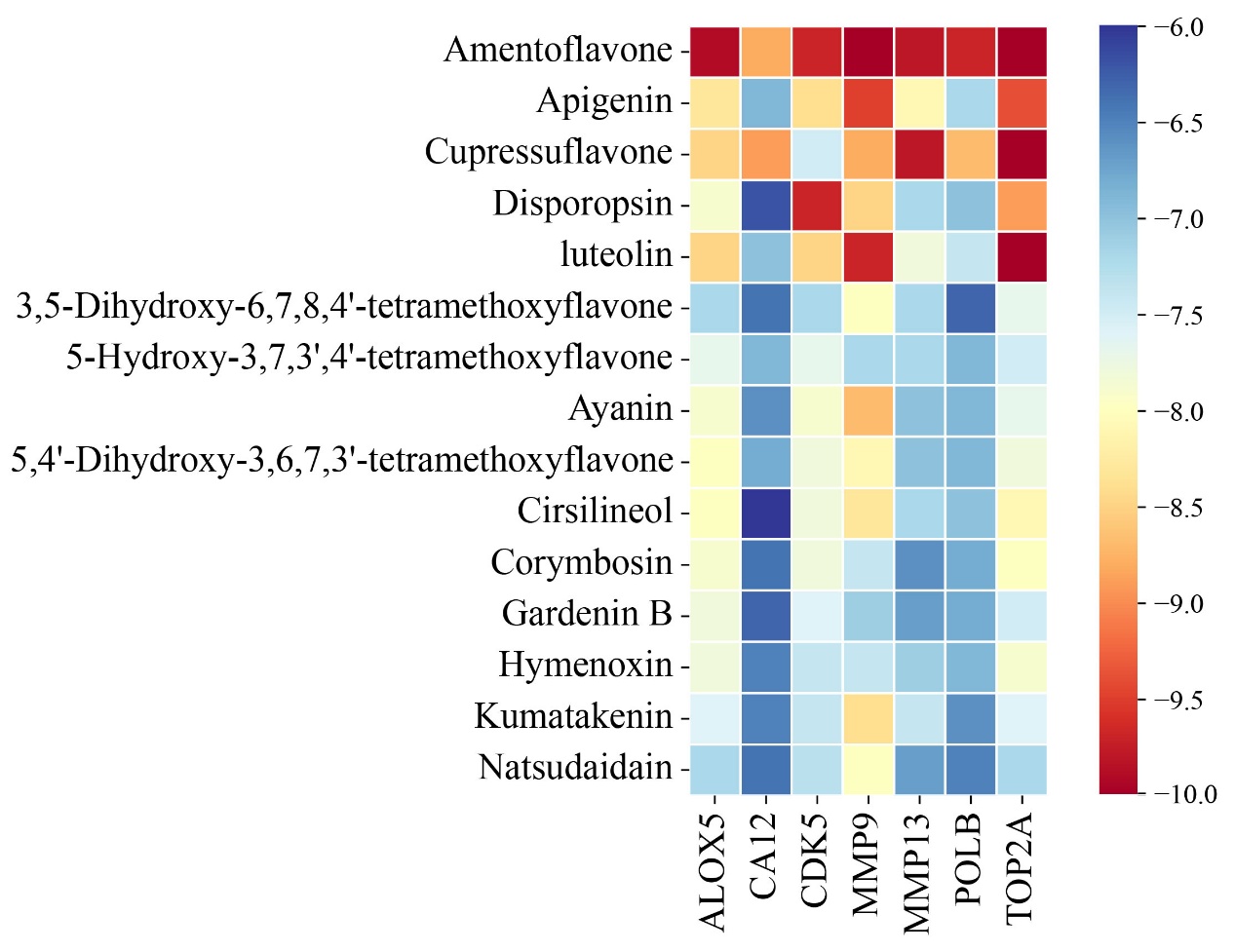


**Fig.S1 Molecular Docking Results.**


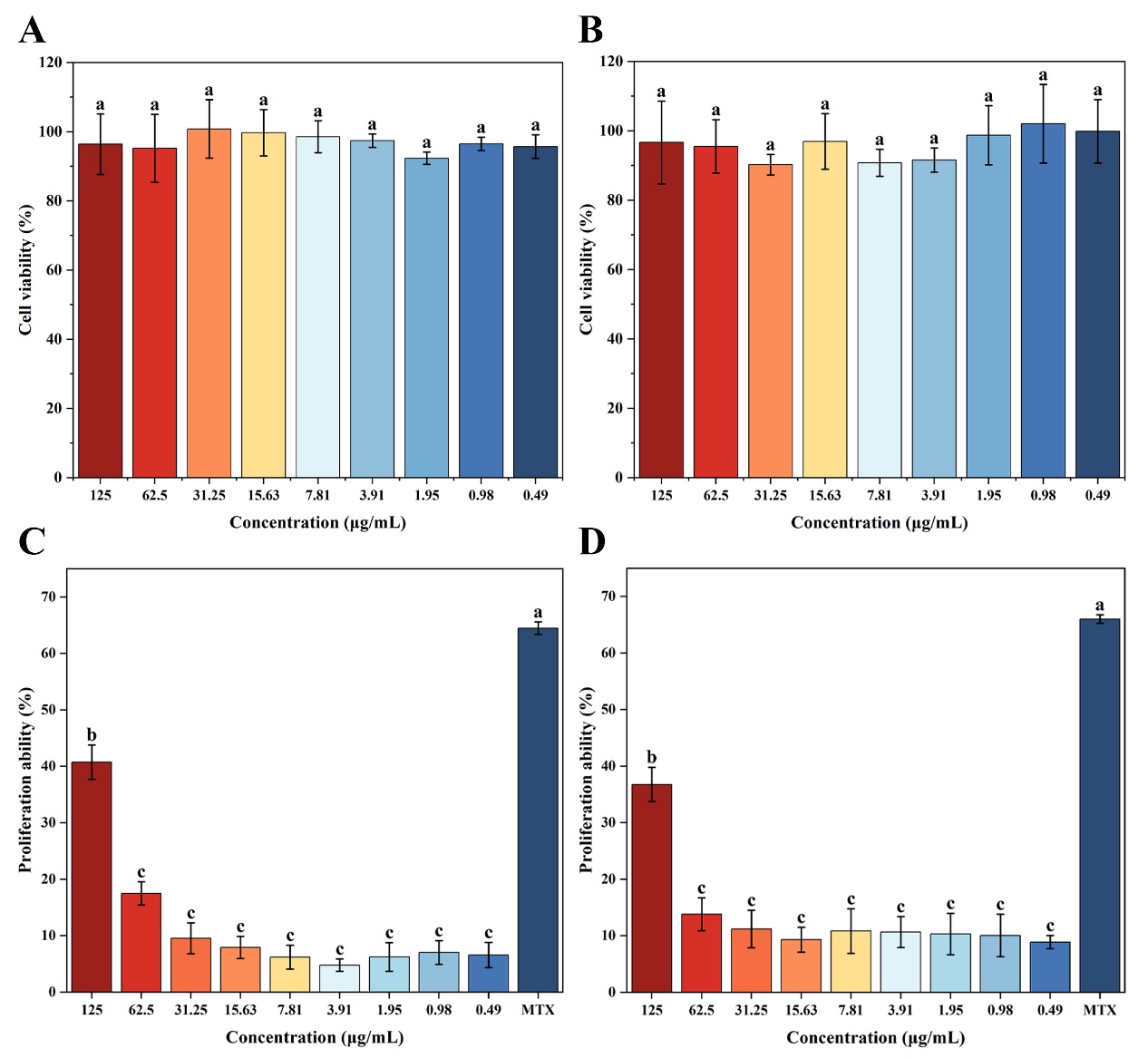


**Fig.S2 GHTFs and AF on the cell viability and proliferation of RAW264.7 cells.** The cell viability of RAW 264.7 treated with (A)GHTFs and (B) AF at various concentrations for 24 h. Different concentrations of (C) GHTFs and (D) AF inhibit the LPS-induced proliferative capacity of RAW 264.7


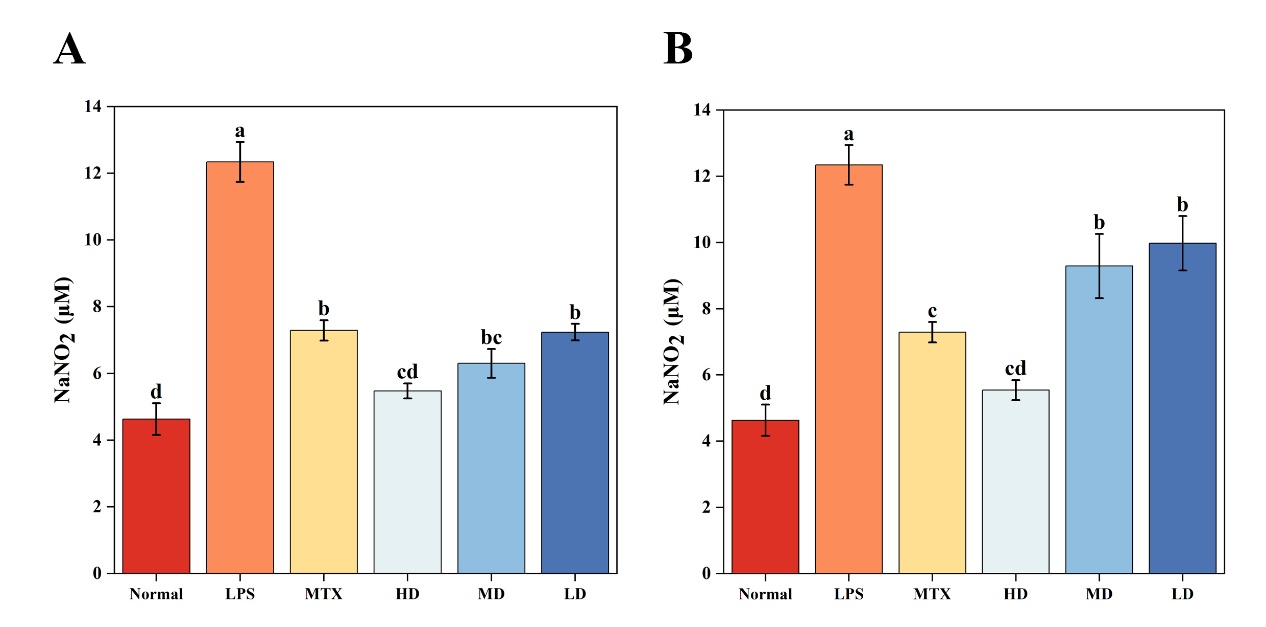


**Fig.S3 Detection of NO content.** (A) GHTFs (B) AF.


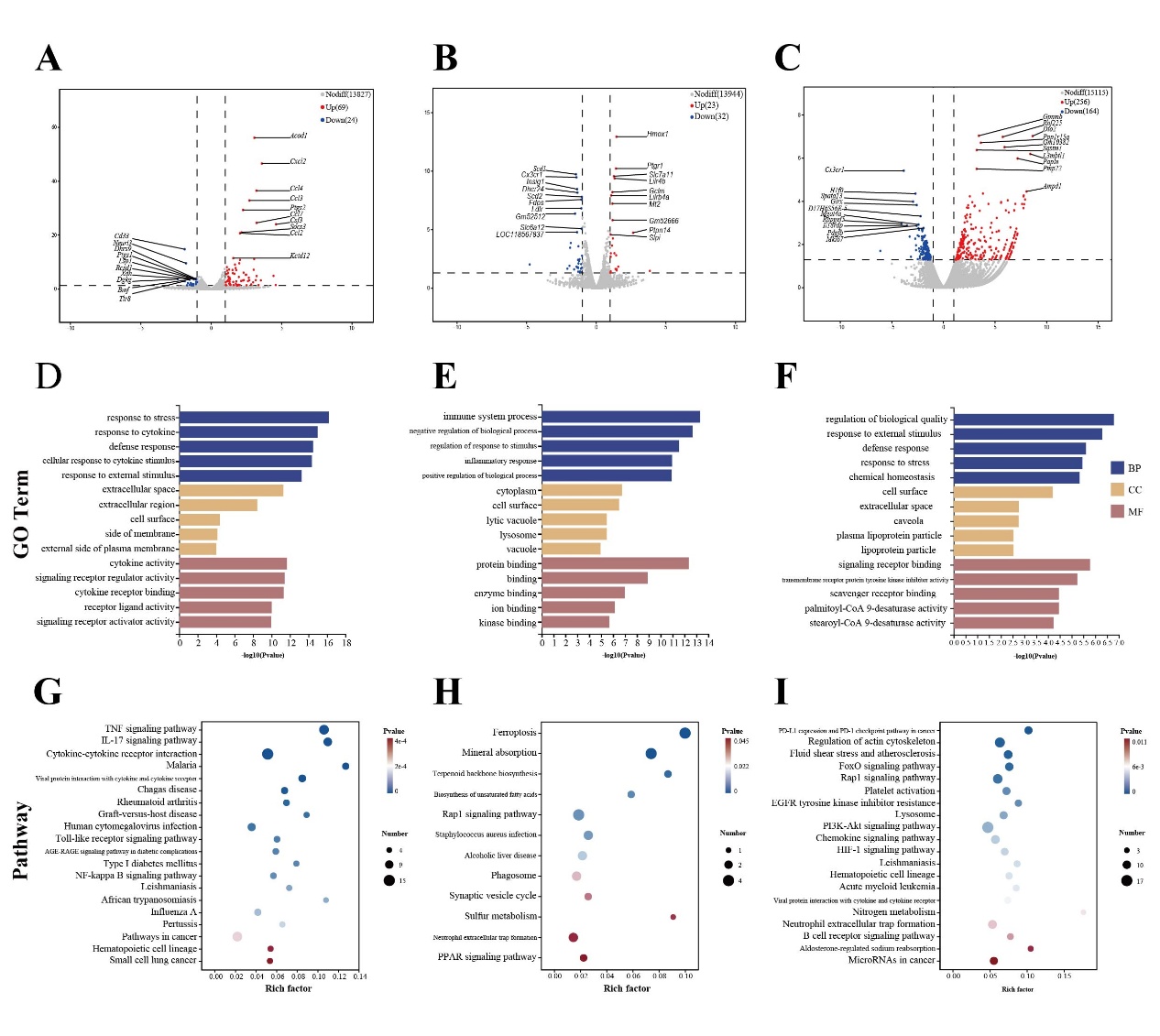
**Fig. S4** **Transcriptomics analysis.** Differential gene volcano map (A) Control v.s. LPS. (B) GHTFs v.s. LPS. (C) AF v.s. LPS. GO enrichment analysis (D) Control v.s. LPS. (E) GHTFs v.s. LPS. (F) AF v.s. LPS. KEGG enrichment analysis (G) Control v.s. LPS. (H) GHTFs v.s. LPS. (I) AF v.s. LPS.


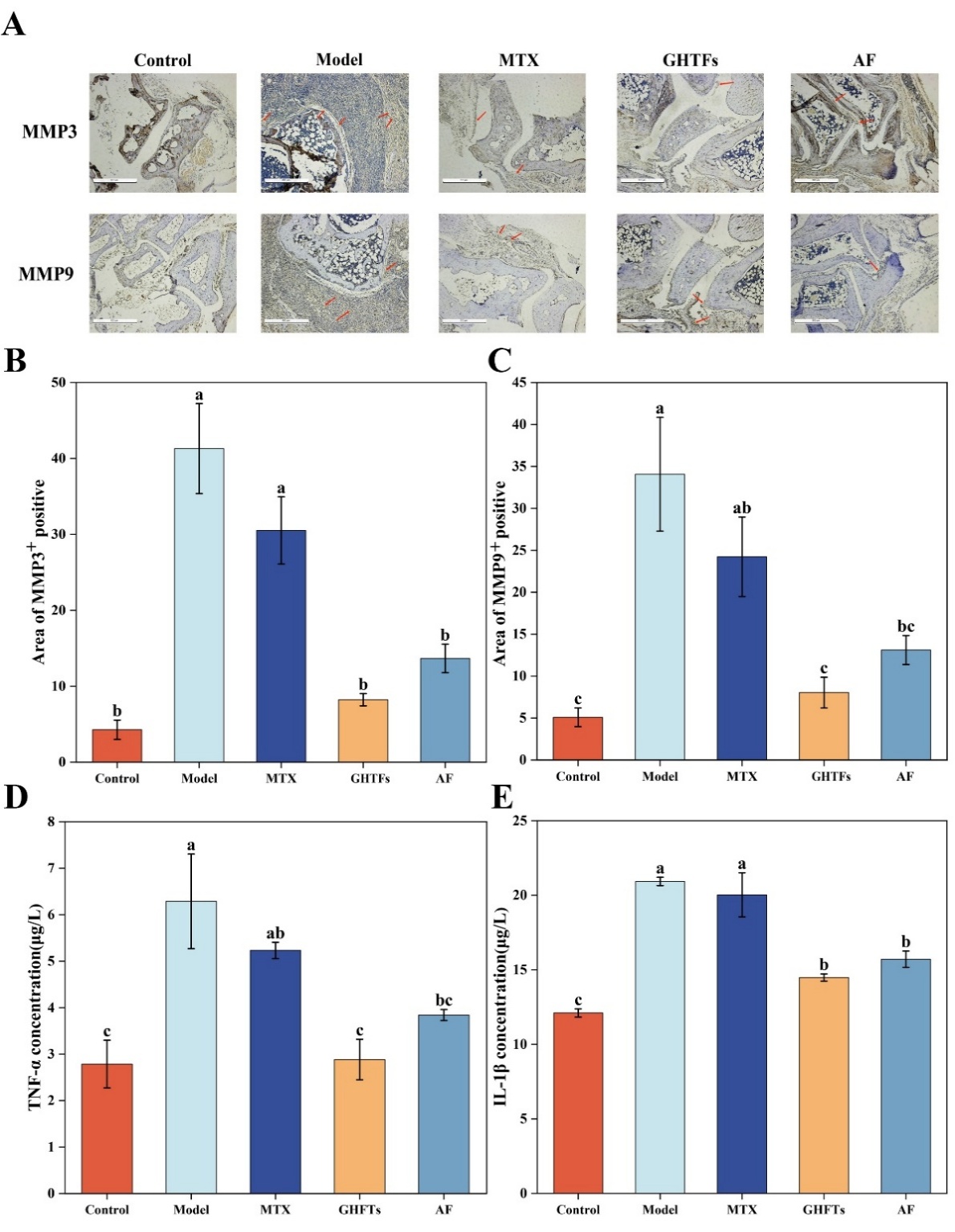


**Fig.S5 The immunohistochemical and enzyme-linked immunosorbent assay.** (A) Representative immunohistochemical staining for MMP3 and MMP9 in synovial sections. Scale bar: 800 μm. (B-C) Quantitative analysis of immunohistochemical staining for MMP3 and MMP9 in synovial tissue. (D-E) The determination of serum pro-inflammatory cytokine concentrations.


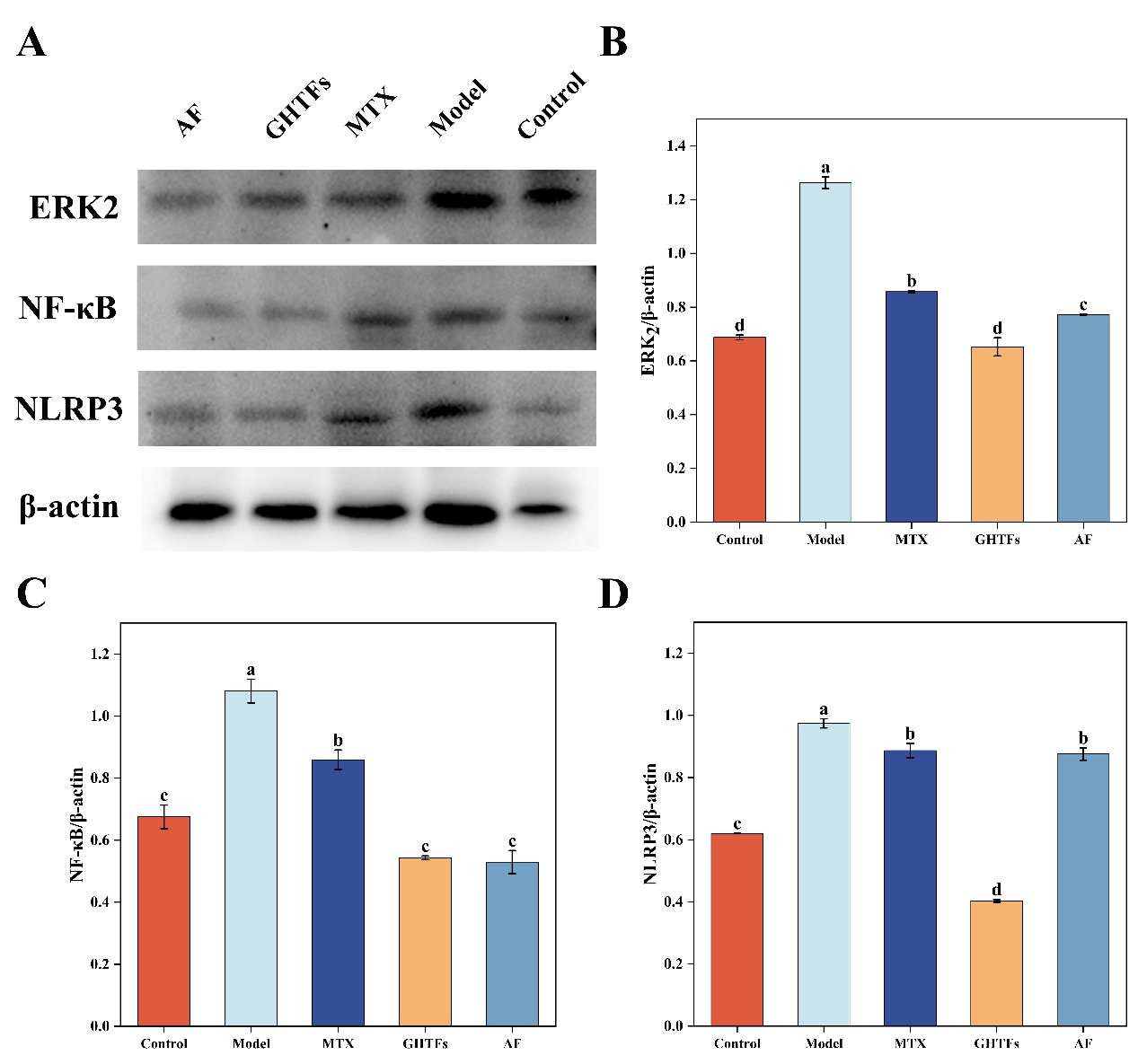
**Fig.S6 The western blot analysis of synovial joint tissues.** (A) The expression analysis of various proteins in synovial tissues of joints. (B) The analysis of reactive optical density values of ERK2 in mice synovial tissues. (C) The analysis of reactive optical density values of NF-κB in mice synovial tissues. (D) The analysis of reactive optical density values of NLRP3 in mice synovial tissues.

**Table S1 Flavonoids information table**

| Compound No. | Compounds | Formula | | | Molecular Weight | |
| --- | --- | --- | --- | --- | --- | --- |
| MOL01 | Gardenin B | |  | C_19_H_18_O_7_ | | 358.11 |
| MOL02 | Corymbosin | |  | C_19_H_18_O_7_ | | 358.11 |
| MOL03 | 5,4'-Dihydroxy-3,6,7,3'-tetramethoxyflavone | |  | C_19_H_18_O_8_ | | 374.10 |
| MOL04 | Ayanin | |  | C_18_H_16_O_7_ | | 344.09 |
| MOL05 | 3,5-dihydroxy-6,7,8, 4'-tetramethoxyflavone | |  | C_19_H_18_O_8_ | | 374.10 |
| MOL06 | Hymenoxin | |  | C_19_H_18_O_8_ | | 374.10 |
| MOL07 | Cirsilineol (4',5-Dihydroxy-3',6,7-trimethoxyflavone) | |  | C_18_H_16_O_7_ | | 344.09 |
| MOL08 | Disporopsin | |  | C_16_H_14_O_6_ | | 302.08 |
| MOL09 | Natsudaidain | |  | C_21_H_22_O_9_ | | 418.13 |
| MOL10 | 5-Hydroxy-3,7,3',4'-tetramethoxyflavone (Retusin) | |  | C_19_H_18_O_7_ | | 358.11 |
| MOL11 | Kumatakenin | |  | C_17_H_14_O_6_ | | 314.08 |
| MOL12 | Cupressuflavone | |  | C_30_H_18_O_10_ | | 538.09 |
| MOL13 | Amentoflavone | |  | C_30_H_18_O_10_ | | 538.09 |
| MOL14 | Apigenin | |  | C_15_H_10_O_5_ | | 270.05 |
| MOL15 | Luteolin | |  | C_15_H_10_O_6_ | | 286.05 |
| MOL16 | Luteolin 7-*O*-glucuronide | |  | C_21_H_18_O_12_ | | 462.08 |
| MOL17 | Scutellarin | |  | C_21_H_18_O_12_ | | 462.08 |
| MOL18 | Hesperetin-7-*O*-glucoside | |  | C_22_H_24_O_11_ | | 464.13 |
| MOL19 | Homoplantaginin | |  | C_22_H_22_O_11_ | | 462.12 |
| MOL20 | Chrysoeriol-7-*O*-Glucoside | |  | C_22_H_22_O_11_ | | 462.12 |
| MOL21 | Quercetin-4'-*O*-glucoside (Spiraeoside) | |  | C_21_H_20_O_12_ | | 464.10 |
| MOL22 | Isohyperoside* | |  | C_21_H_20_O_12_ | | 464.10 |
| MOL23 | Quercetin-3-*O*-glucoside （isoquercitrin) | |  | C_21_H_20_O_12_ | | 464.10 |
| MOL24 | Diosmetin-3'-*O*-galactoside* | |  | C_22_H_22_O_11_ | | 462.12 |
| MOL25 | 6-*C*-MethylKaempferol-3-glucoside | |  | C_22_H_22_O_11_ | | 462.12 |
| MOL26 | Quercetin-7-*O*-(6''-malonyl)glucoside | |  | C_24_H_22_O_15_ | | 550.10 |
| MOL27 | Yuanhuanin | |  | C_22_H_22_O_11_ | | 462.12 |
| MOL28 | Apigenin-7-*O*-glucuronide | |  | C_21_H_18_O_11_ | | 446.09 |
| MOL29 | Quercetin-4'-*O*-glucuronide | |  | C_21_H_18_O_13_ | | 478.08 |
| MOL30 | Gossypetin-3-*O*-(6''-malonyl)glucoside | |  | C_24_H_22_O_16_ | | 566.09 |
| MOL31 | Cyanidin-3-*O*-(6''-*O*-caffeoyl)glucoside | |  | C_30_H_27_O_14+_ | | 611.14 |
| MOL32 | Myricetin-3-*O*-(6''-malony)glucoside | |  | C_24_H_22_O_16_ | | 566.09 |
| MOL33 | 6-Hydroxyluteolin 5-glucoside | |  | C_21_H_20_O_12_ | | 464.10 |
| MOL34 | Tetahydroxyflavone-7-*O*-glucuronide | |  | C_21_H_18_O_12_ | | 462.08 |
| MOL35 | Quercetagetin-7-*O*-glucoside | |  | C_21_H_20_O_13_ | | 480.09 |
| MOL36 | Kaempferol-3-*O*-glucuronide | |  | C_21_H_18_O_12_ | | 462.08 |
| MOL37 | 3',5',5,7-Tetrahydroxy-4'-methoxyflavanone-3'-*O*-glucoside | |  | C_22_H_24_O_12_ | | 480.13 |

**Table S2 Arthritis scoring criteria**

| Sore | Grading scheme |
| --- | --- |
| 0 | Without any macroscopic signs of arthritis |
| 1 | Minor redness and swelling of the ankle or significant redness and swelling of individual claw toes |
| 2 | Moderate redness and swelling of the ankle or wrist joints |
| 3 | Severe redness and swelling of the entire paw and claw toes |
| 4 | Polyarticular joint swelling |
